# TRA2A negatively regulates HIV-1-induced macrophage pyroptosis by mediating TXNIP expression in an m6A-dependent manner

**DOI:** 10.1038/s41420-026-03236-2

**Published:** 2026-06-26

**Authors:** Xing Tao, Jinming Su, Tongxue Qin, Xiu Chen, Rongfeng Chen, Yinlu Liao, Ran Duan, Shiyi Lai, Youjin Huang, Jinmiao Li, Li Ye, Hao Liang, Junjun Jiang

**Affiliations:** 1 Guangxi Key Laboratory of AIDS Prevention and Treatment, School of Public Health, Guangxi Medical University, Nanning, China; 2 China (Guangxi) - ASEAN Joint Laboratory of Emerging Infectious Diseases, Life Sciences Institute, Guangxi Medical University, Nanning, China; 3 Department of Maternity-Child Health and Family Planning Services, Nanning Maternity and Child Health Hospital Affiliated to Youjiang Medical University for Nationalities, Nanning, China

**Keywords:** Viral infection, Infection

## Abstract

Human Immunodeficiency Virus (HIV) infection remains a global health challenge, with the roles of N6-methyladenosine (m6A) RNA modification and Transformer 2 Alpha Homolog (TRA2A) in viral regulation yet to be fully elucidated; this study aimed to investigate how TRA2A-mediated m6A modification of Thioredoxin Interacting Protein (TXNIP) regulates macrophage pyroptosis during HIV-1 infection. Lentiviral-mediated knockdown and overexpression of TRA2A were employed to evaluate its effects on TXNIP expression, m6A modifications, and HIV-1 replication, while pyroptosis and inflammatory responses were assessed through LDH release assays, cytokine measurements, and analysis of the NLRP3/Caspase-1/GSDMD signaling pathway. Results showed significant downregulation of TRA2A in macrophages from HIV-1 patients, and TRA2A knockdown promoted viral replication while activating NLRP3/Caspase-1/GSDMD-mediated pyroptosis; mechanistically, TRA2A recognized m6A sites on TXNIP mRNA to reduce its stability, thereby inhibiting inflammasome activation, with these effects reversed by TRA2A overexpression or TXNIP/pyroptosis inhibitors, confirming TRA2A’s critical role in balancing antiviral defense and inflammation control. Collectively, TRA2A functions as an m6A regulator to balance antiviral defense and inflammation via the TXNIP-mediated NLRP3 pathway, establishing a novel regulatory axis and highlighting TRA2A as a potential dual-target therapeutic candidate.

## Introduction

HIV, the causative agent of Acquired Immunodeficiency Syndrome (AIDS), is one of the most extensively studied pathogens globally [1]. Since its identification in the early 1980s, HIV/AIDS has reached pandemic proportions, with over 39.9 million individuals reported to be living with the virus worldwide and 630,000 AIDS-related deaths recorded in 2023 alone [2]. HIV-1 infection exhibits substantial interindividual variability. Among HIV-1-infected individuals not receiving antiretroviral therapy (ART), approximately 80% progress to the AIDS stage after an asymptomatic period of 8–10 years. This progression is characterized by a persistent decline in CD4^+^ T-cell counts to below 200 cells/μL and progressive impairment of immune function, and these individuals are defined as HIV-1 typical progressors (TPs) [3]. Since the 1980s, antiretroviral therapy (ART) has transformed HIV-1 infection into a manageable chronic condition, yet it fails to eradicate the virus or fully restore immune function. While ART improves certain immunological parameters, its efficacy in reducing pro-inflammatory cytokines like IL-1β remains controversial, as their levels stay elevated vs. uninfected controls. Therefore, there is an urgent need to develop novel therapeutic strategies aimed at eradicating the virus or fully mitigating its immunopathological effects.

Human immunodeficiency virus type 1 (HIV-1) primarily targets CD4^+^ T lymphocytes and macrophages, two cell populations critical for orchestrating antiviral immune responses [4]. While CD4^+^ T cell depletion remains a hallmark of disease progression, emerging evidence underscores macrophages as pivotal players in sustaining viral persistence and immunopathology [5]. Unlike circulating lymphocytes, tissue-resident macrophages exhibit extraordinary plasticity in modulating innate immune defenses against HIV-1 through phagocytosis, cytokine production, and antigen presentation [6]. Paradoxically, these frontline defenders become viral reservoirs and amplifiers upon infection, with HIV-1-induced pyroptosis representing a key mechanism linking cellular demise to systemic inflammation [7]. This lytic cell death modality triggers the release of proinflammatory cytokines that paradoxically enhance viral dissemination while depleting immune effector populations [8]. Identifying endogenous factors capable of mitigating HIV-1-associated pyroptosis thus holds promise for breaking this vicious cycle.

In recent years, the rapid development of the emerging field of “epitranscriptomics” has provided innovative perspectives and exploratory pathways for HIV/AIDS treatment strategies [9]. Epitranscriptomics focuses on the regulation of heritable gene expression without altering the underlying gene sequence [10]. Among various RNA modifications, m6A has emerged as a critical modulator of host-virus interactions. As the most abundant internal mRNA modification in eukaryotes [11], m6A dynamically regulates RNA metabolism processes essential for viral replication, including splicing, stability, and translation [12]. Conversely, certain m6A writers may restrict viral replication by depositing methyl groups on proviral DNA [13]. This duality positions m6A modifiers as potential therapeutic targets requiring precise functional characterization.

In this study, MeRIP-seq analysis was performed on CD4^+^positive cells from TP group, with healthy individuals serving as controls. This analysis identified a group of m6A-related differential genes associated with HIV-1 infection and replication. Among these, the m6A regulatory factor TRA2A was significantly downregulated in the TP group. Research by Sanqi An’s team [14] was the first to reveal that TRA2A interacts with m6A writers, selectively promotes methylation at m6A sites, and co-localizes with RNA-binding targets. Studies on avian influenza viruses (IAVs) have shown that TRA2A can inhibit the replication of avian-derived IAVs by binding to intronic splicing silencer motifs within viral mRNA, while promoting the replication of human-derived IAVs [15]. Regarding the role of TRA2A in HIV-1 infection, only one study has explored the differential effects of the two isoforms, Tra2α and Tra2β, on HIV-1 RNA processing and expression. This study demonstrated that overexpression of either Tra2α or Tra2β reduces the expression of HIV-1 Gag and Env proteins [16]. However, the specific mechanisms by which TRA2A influences HIV-1 infection remain largely unexplored. Investigating the effects of TRA2A knockdown or overexpression on HIV-1 replication, as well as elucidating how these changes affect host cell responses to HIV-1 infection, may provide valuable insights. Such studies could identify TRA2A as a potential target for the development of novel antiviral therapeutic strategies.

Based on the findings, this study identified TRA2A as a key m6A regulatory factor associated with TPs. The expression levels of TRA2A were first characterized in human samples. Subsequently, a TRA2A-knockdown macrophage model was established using the THP-1 cell line to investigate the role and mechanisms of TRA2A as an m6A regulator in modulating immune responses during HIV-1 infection in macrophages.

## Results

### HIV-1 Infection Leads to downregulated TRA2A Expression in Macrophages

**Fig. 1:**
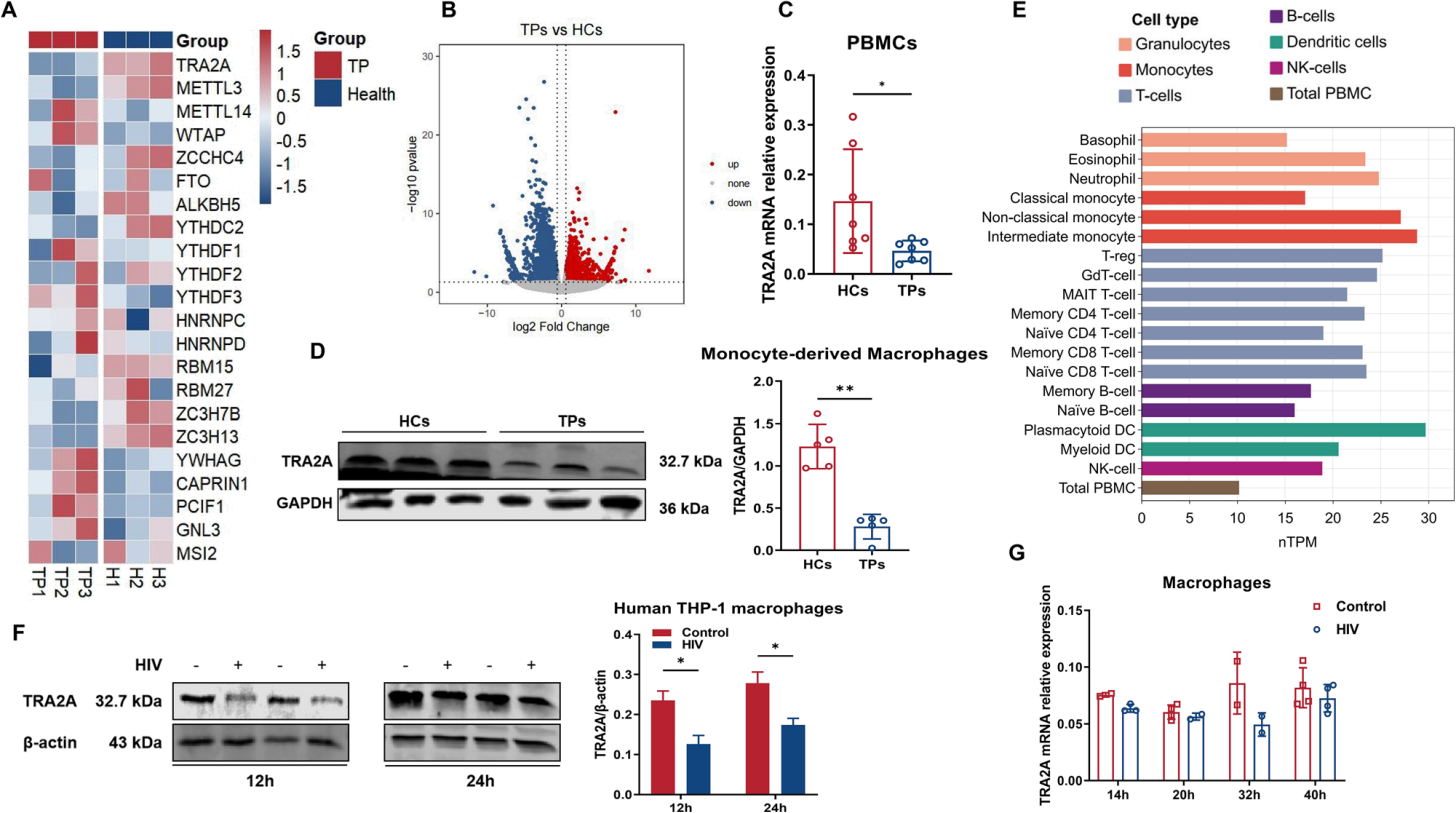
HIV-1 infection is associated with reduced TRA2A expression in macrophages. **A**, **B** Heatmap and volcano plot of mRNA expression levels of m6A regulators in TP population and HC population, showing the mRNA expression levels of each m6A regulator, where red indicates up - regulation and blue indicates down - regulation; **C** Expression level of TRA2A in PBMCs of TP population and HC population; **D** Protein level expression of TRA2A in TPs and HCs after matching; **E** mRNA expression level of TRA2A in various immune cells; **F** The protein expression level of TRA2A was down-regulated in the THP-1-derived macrophage group infected with HIV-1, and the difference was statistically significant; **G** The mRNA expression level of TRA2A in macrophages derived from the peripheral blood of healthy people infected with HIV-1 showed a downward trend. *P < 0.05, **P < 0.01, ***P < 0.001.

This study aimed to identify differential m⁶A regulatory factors in TPs. Per established inclusion/exclusion criteria, 6 individuals were enrolled (3 untreated TP patients, 3 HCs). CD4-positively selected cells, which predominantly encompass CD4^+^ T cells, macrophages, and monocytes, were subjected to MeRIP-seq analysis to elucidate the mRNA expression profiles of m6A methylation factors between the two groups. As shown in Fig. 1A, B, the findings revealed a statistically significant disparity in the mRNA expression levels of TRA2A, an m6A regulatory factor, between the TPs and the HCs (*P* < 0.05). To explore TRA2A association with HIV-1 infection, additional TP and HC cohorts were subsequently enrolled post-MeRIP-seq and bioinformatics analysis. qPCR of PBMC-derived macrophages revealed 3.5-fold downregulated TRA2A mRNA in TP versus HC, consistent with sequencing data (Fig. 1C). Proteinatlas database analysis (Fig. 1E) demonstrated immune-specific TRA2A overexpression in monocytes. Given the classification of target cells for HIV-1 viral replication, further analysis was conducted to assess TRA2A expression within macrophages sourced from human peripheral blood. Upon comparison of TRA2A protein expression levels between TPs and HCs, it was observed that the TRA2A protein levels in macrophages originating from the peripheral blood of individuals with typical progression were downregulated by a factor of 4.2 relative to the healthy controls, as in Fig. 1D.

To elucidate the alterations in TRA2A expression within macrophages during HIV-1 infection, two distinct macrophage infection models were established: one utilizing Monocyte-derived Macrophages (MDMs) from healthy donors, and the other using THP-1 cell line-derived macrophages (TDMs). MDMs were generated by inducing the differentiation of PBMCs with GM-CSF. TDMs were obtained by treating THP-1 cells with PMA to induce macrophage differentiation. Following a 12-h infection with HIV-1 Bal, cultures were extensively washed with PBS to remove unbound virus and then maintained in culture. At specified intervals, samples were collected for RNA and protein harvesting to assess TRA2A mRNA and protein expression levels, as presented in Fig. 1F. MDMs showed decreased TRA2A mRNA post-infection; TRA2A protein was significantly downregulated 1.91-fold (12 h) and 1.58-fold (24 h) (Fig. 1G). These data confirm HIV-1 infection-associated TRA2A downregulation in macrophages, validating clinical observations.

### TRA2A inhibits HIV-1 replication in macrophages

**Fig. 2:**
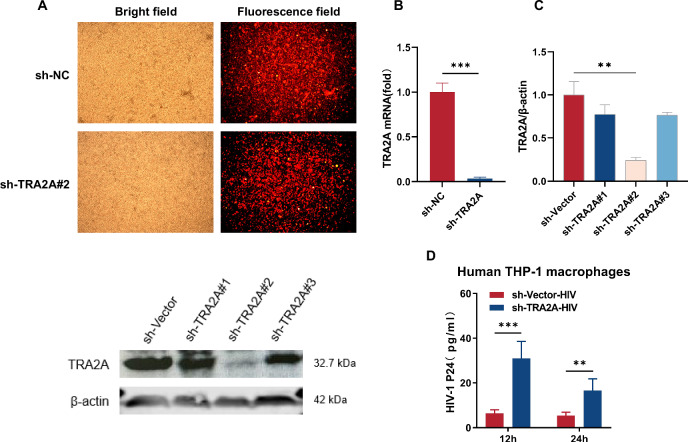
TRA2A inhibits HIV-1 replication in macrophages. **A** Construction of TRA2A - knockdown macrophage cell line derived from THP-1, confirming the intensity of RFP fluorescence; **B** qPCR was used to detect the mRNA expression level of TRA2A in shNC and shTRA2A cells; **C** WB was used to detect the protein expression level of TRA2A in shNC and shTRA2A cells. **D** Detection of HIV-1 p24 antigen showed that TRA2A inhibited the replication of HIV-1 virus in macrophages. *P < 0.05, **P < 0.01, ***P < 0.001.

To ascertain the role of TRA2A in conferring resistance to HIV-1 infection in macrophages, a lentiviral-mediated knockdown approach was employed to generate TRA2A knockdown cell lines in THP-1 cells. Following selection with neomycin, stable cell lines were established, and the enhancement of RFP expression was observed under a fluorescence microscope, as depicted in Fig. 2A. Upon neomycin selection, there was a marked increase in the proportion of RFP-expressing cells. The efficiency of transduction in the TRA2A knockdown macrophages was further validated using qPCR and WB analysis. qPCR analysis revealed a 97% reduction in TRA2A mRNA levels in knockdown cells relative to the control knockdown group, and WB analysis demonstrated an approximate 80% decrease in sh-TRA2A-#2 protein levels (Fig. 2B, C), confirming the successful establishment of the TRA2A knockdown cell line. Subsequently, the THP-1-derived macrophages with TRA2A knockdown were induced to differentiate, and both knockdown and control groups were infected with the HIV-1 Bal. At various time points post-infection, cell supernatants were harvested and the levels of HIV-1 p24 protein were measured using ELISA, as shown in Fig. 2D. The data demonstrated that in cells with TRA2A knockdown infected by HIV-1 Bal, the HIV-1 p24 protein levels were significantly elevated by 4.88-fold and 3.11-fold at 12 and 24 h post-infection, respectively, compared to the shNC-infected control group, with both increases being statistically significant (*P* < 0.05).

Collectively, these findings indicate that macrophages with TRA2A knockdown exhibit heightened levels of HIV-1 p24 protein upon infection, suggesting that TRA2A may play a role in suppressing HIV-1 replication within macrophages, thereby exerting an anti-HIV-1 effect.

### Correlative analysis of mRNA-m6A methylation profiling between TPs and HCs

**Fig. 3:**
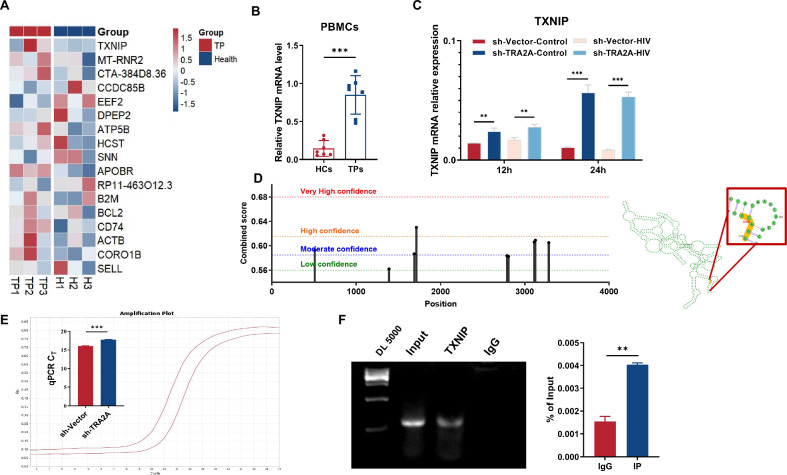
TRA2A regulates TXNIP expression through m6A modification and direct RNA binding. **A** Heatmap of mRNA - m6A methylation correlation analysis, where red indicates up - regulation and blue indicates down - regulation; **B** The mRNA level of TXNIP is up - regulated in the population with typical progression of HIV-1 infection; **C** Knockdown of TRA2A up-regulates the mRNA expression level of TXNIP both in the case of HIV-1 infection and without HIV-1 infection; **D** SRAMP predicts the distribution of prediction scores at different positions of TXNIP; **E** The SELECT experiment verifies that there is an m6A site on TXNIP regulated by TRA2A; **F** The binding of TRA2A and TXNIP is verified by the RIP experiment. *P < 0.05, **P < 0.01, ***P < 0.001.

In the context of our study, the replication of HIV-1 is observed to be modulated by intricate innate immune signaling cascades. To delineate the antiviral mechanism of TRA2A against HIV-1, an interaction heatmap of differentially expressed mRNA-m6A genes at the protein level between the Hcs and TPs groups was constructed using the STRING database. This analysis aimed to filter and identify potential key target genes. The differential mRNA-m6A genes identified between the two groups include TXNIP, mitochondrially encoded 16S ribosomal RNA (MT-RNR2), uncharacterized long non-coding RNA CTA-384D8.36 (CTA-384D8.36), coiled-coil domain containing 85B (CCDC85B), eukaryotic translation elongation factor 2 (EEF2), dipeptidase 2 (DPEP2), ATP synthase F1 subunit beta (ATP5B), hematopoietic cell signal transducer (HCST), stannin (SNN), apolipoprotein B receptor (APOBR), uncharacterized long non-coding RNA RP11-463O12.3 (RP11-463O12.3), beta-2-microglobulin (B2M), B-cell lymphoma 2 (BCL2), CD74 molecule (CD74), actin beta (ACTB), coronin 1B (CORO1B), and selectin L (SELL), among others (Fig. 3A). To substantiate the findings from the pathway enrichment analysis, the expression levels of these differential factors within PBMCs of HIV-1-infected typical progressors and healthy individuals were assessed using qPCR. Notably, TXNIP, a protein known to interact with thioredoxin, exhibited a 6.07-fold increase in mRNA expression in the typical progressors compared to the HCs (Fig. 3B), with this difference being statistically significant (*P* < 0.05). In contrast, the expression levels of other differentially expressed genes did not present significant disparities between the two groups. Further analysis of TXNIP mRNA expression post-infection revealed that in TRA2A knockdown macrophages infected with HIV-1, the expression of TXNIP was upregulated by 1.68-fold and 6.25-fold at 12 and 24 h, respectively, compared to the control infected group, with these differences also reaching statistical significance (Fig. 3C). Collectively, these observations suggest a correlation between TXNIP and HIV-1 infection, and indicate a regulatory role for TRA2A on TXNIP expression.

### Prediction and Validation of m^6^A Modification Sites on TXNIP Regulated by TRA2A

Given that TRA2A is a m6A regulator, the hypothesis that TRA2A may regulate TXNIP protein synthesis by mediating m6A modification on TXNIP, thereby affecting downstream signaling pathways, was explored. To investigate this, the online database SRAMP was utilized to predict the presence of m6A single-base sites on TXNIP. The cDNA sequence of the human TXNIP gene was downloaded from NCBI and subsequently entered into the SRAMP database. The prediction of m6A sites and secondary structure was then conducted. The findings revealed the existence of m6A modification sites on TXNIP, with a high-confidence m6A modification site identified at position 274. The secondary structure prediction also indicated the presence of an m6A modification site (Fig. 3D). Primers were designed for TXNIP using this site, and the SELECT experiment was used to verify whether TXNIP has a target site for TRA2A. The results showed that there was a difference in m6A abundance at a specific site of TXNIP between the sh-TRA2A group and the sh-Vector group. Combined with the fluorescence amplification curve and qPCR CT values, there was a statistical difference between the two groups (Fig. 3E), which fully explains that TXNIP has a target site for TRA2A, consistent with the above bioinformatics prediction. It is clear that in THP-1 cells, TRA2A can mediate the m6A modification of TXNIP (Fig. 3F). Further RIP experiments were conducted between TRA2A and TXNIP, and the results showed that there is an RNA-protein interaction between TRA2A and TXNIP.

### TRA2A regulates pyroptosis and inflammatory responses in macrophages following HIV-1 infection

Although our preliminary research has identified that TRA2A influences the expression of TXNIP through the modulation of m6A methylation, the precise mechanism by which the signal is conveyed from the nucleus to the cytoplasm and subsequently impacts HIV-1 viral replication remains a scientific question of interest within our study. During the cultivation of TRA2A knockdown THP-1 cell lines, an increased number of shTRA2A cell deaths and a more rapid rate of cell death compared to shNC cells were observed. Given the existing literature on TXNIP, which implicates this protein in oxidative stress and inflammatory responses, it is known to activate the NLRP3 inflammasome complex via endoplasmic reticulum stress, initiate mitochondrial stress-induced pyroptosis, and stimulate the release of inflammatory cytokines. Consequently, we propose a scientific hypothesis that TRA2A may modulate the interaction between TXNIP and NLRP3, leading to the induction of pyroptosis in macrophages and, in turn, mediating a more distal effect on viral replication.

**Fig. 4:**
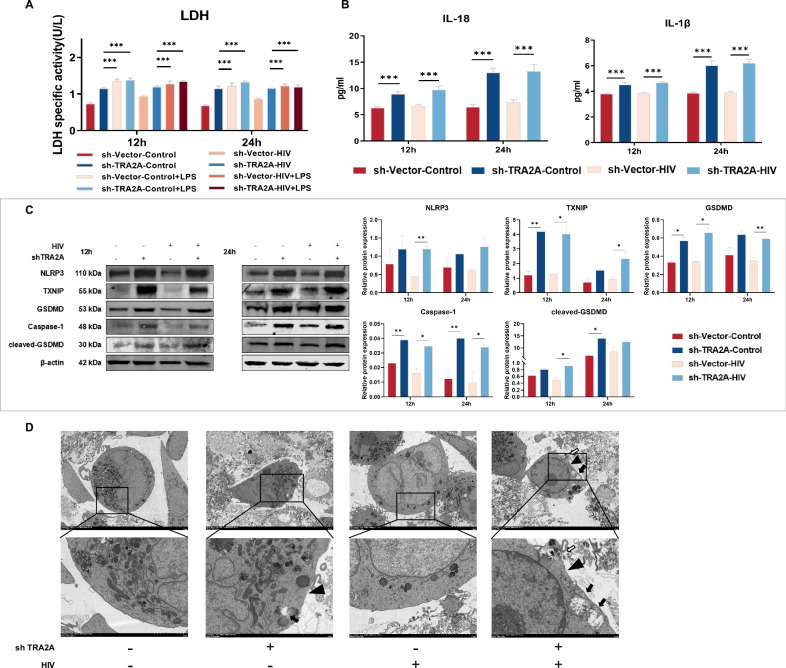
Changes in pyroptosis markers and inflammatory levels after TRA2A knockdown in THP-1-derived macrophages. **A** LPS was used to mimic the inflammatory environment for pyroptosis; knockdown of TRA2A and LPS treatment both enhanced LDH activity in the presence or absence of HIV-1 infection; **B** Knockdown of TRA2A promoted the secretion of inflammatory cytokines IL-18 and IL-1β in both HIV-1-infected and uninfected conditions; **C** TRA2A knockdown activated the NLRP3/Caspase-1/GSDMD pyroptosis pathway in THP-1-derived macrophages after HIV-1 infection. **D** Transmission electron microscopy(TEM) images showing the sh-Vector-Control group exhibit intact plasma membranes and normal morphology. The sh-Vector group infected with HIV-1 displays mild early signs of cellular stress. The sh-TRA2A-Control and sh-TRA2A-HIV groups exhibited typical characteristics of pyroptosis. Black arrows indicate vacuolization of organelles, white arrows indicate cell membrane rupture, and black triangles indicate the formation of pyroptotic pores (Low magnification: × 2.0k, high magnification: × 8.0k). *P < 0.05, **P < 0.01, ***P < 0.001.

The secretion of LDH is considered one of the markers of inflammatory cell death. LPS induces an inflammatory response in macrophages and can serve as a positive control group for pyroptosis. We first treated TRA2A knockdown THP-1 cell strains with 1 μg/mL LPS for 24 h, then detected the release of LDH in the cell culture supernatant. As shown in Fig. 4A, compared with the control group sh-Vector-HIV, both sh-Vector-HIV-LPS and sh-TRA2A-HIV groups showed a significant increase in LDH release. Subsequently, we detected the secretion of the key pro-inflammatory cytokines IL-18 and IL-1β in the process of pyroptosis, using TRA2A knockdown/negative control THP-1 cells, infected/uninfected with HIV-1 Bal virus for 12 h, and then detected the expression of IL-18 and IL-1β. The results showed that the expression of IL-18 and IL-1β was stably upregulated at multiple time points after TRA2A knockdown, with a statistically significant difference (*P* < 0.05) (Fig. 4B), which preliminarily indicates that TRA2A knockdown can promote macrophage pyroptosis. We further detected the differences in protein expression levels of key factors in the GSDMD/Caspase-1 pathway between the infected group and the control group at 12 h and 24 h after TRA2A knockdown or control in THP-1 cells. After TRA2A knockdown macrophages were infected with HIV-1, the protein levels of TXNIP, NLRP3, pyroptosis-related protein GSDMD, cleaved-GSDMD, and the effector enzyme Caspase-1 were all significantly upregulated compared to the control infected group, and it is noteworthy that the change in the cleavage of GSDMD further confirmed the occurrence of pyroptosis (Fig. 4C). To provide direct ultrastructural evidence for the occurrence of pyroptosis, we performed TEM on THP-1-derived macrophages. As shown in Fig. 4D, cells in the uninfected control groups(sh-Vector-Control) maintained normal cellular morphology with intact continuous plasma membranes and dense, well-structured cytoplasm. Following HIV-1 infection alone(sh-Vector-HIV), the macrophages exhibited early signs of cellular stress, characterized by mitochondrial condensation and increased cytoplasmic vacuolization, yet the plasma membrane integrity remained preserved. In stark contrast, TRA2A knockdown cells exhibited classical features of pyroptosis, which were further exacerbated by HIV infection. In sh-TRA2A-Control group, a small proportion of cells displayed morphological hallmarks of pyroptosis, characterized by compromised plasma membrane integrity and large translucent vacuoles at the cell periphery. Strikingly, the incidence of pyroptosis was significantly elevated in the sh-TRA2A-HIV group, with overt plasma membrane discontinuity and rupture observed, leading to the leakage of cytoplasmic contents into the extracellular space. Concurrently, the formation of pyroptotic pores was readily detectable in these cells.

### TRA2A targets TXNIP to regulate macrophage pyroptosis and inflammatory responses following HIV-1 infection

**Fig. 5:**
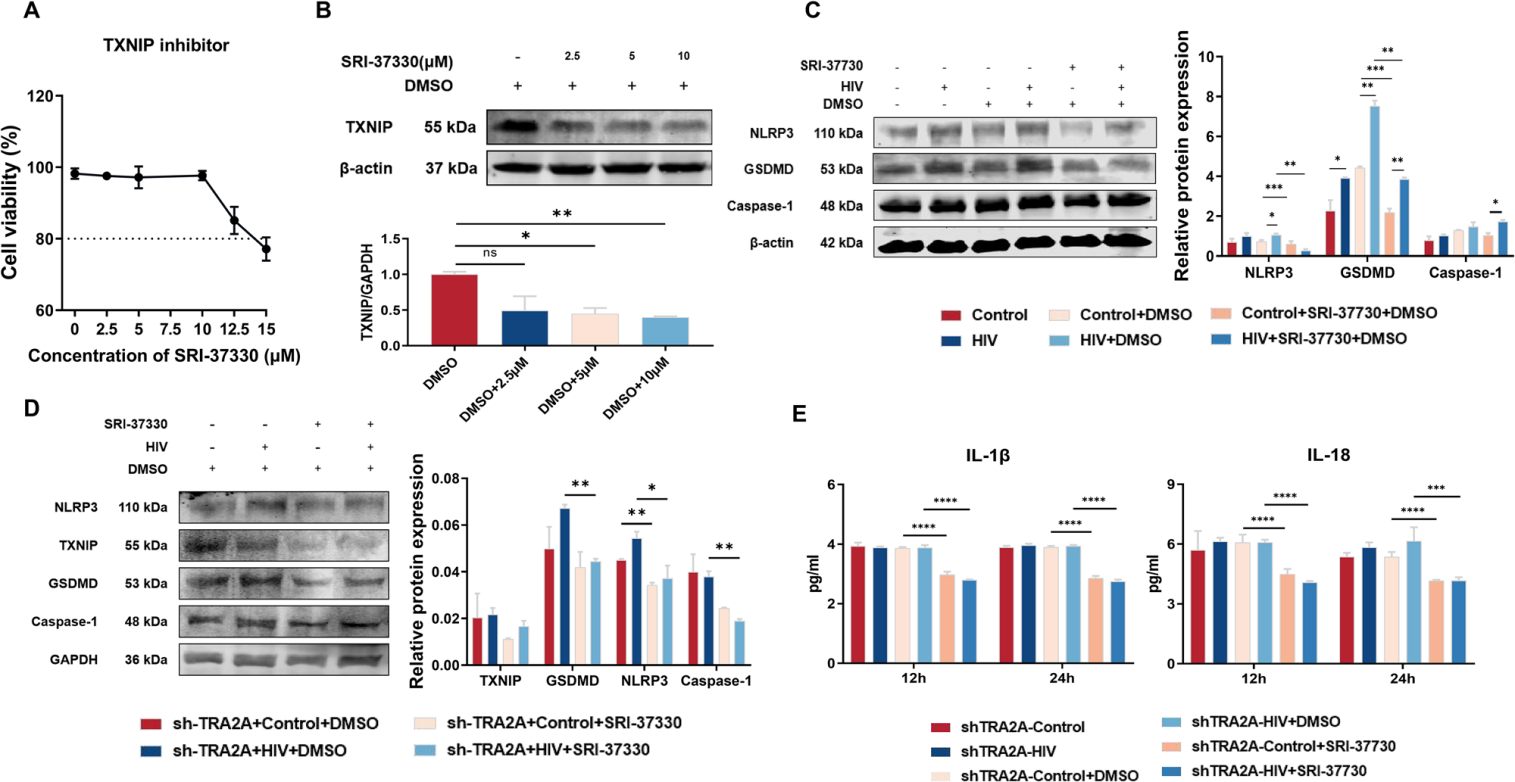
TXNIP inhibition attenuates TRA2A knockdown-induced activation of macrophage pyroptosis and inflammatory responses. **A** CCK-8 cell viability assay was used to assess the effect of SRI-37330 reagent (dissolved in DMSO) on THP-1 cell viability; **B** WB was performed to detect the inhibitory effect of TXNIP inhibitor at different concentrations; **C** Inhibition of the NLRP3/Caspase-1/GSDMD pyroptosis pathway in THP-1-derived macrophages treated with TXNIP inhibitor; **D** TXNIP inhibitor suppressed the activation of NLRP3/Caspase-1/GSDMD pyroptosis pathway in TRA2A-knockdown THP-1-derived macrophages after HIV-1 infection; **E** TXNIP inhibitor inhibited the up-regulated secretion of inflammatory cytokines IL-18 and IL-1β in TRA2A-knockdown THP-1-derived macrophages after HIV-1 infection.

To explore the interaction between TRA2A and TXNIP, and their downstream effects, TXNIP inhibitor SRI-37330 was used to assess how TXNIP downregulation impacts the NLRP3/Caspase-1/GSDMD cascade. First, CCK-8 assay evaluated viability of THP-1 cells treated with DMSO-dissolved SRI-37330. The findings demonstrated that SRI-37330 concentrations ranging from 2.5 to 10 μmol/L were non-toxic to THP-1 cells (Fig. 5A). Next, THP-1 cells were pre-treated with SRI-37330 (2.5/5/10 μmol/L) before HIV-1 Bal infection to verify TXNIP suppression efficacy (Fig. 5B). Maximal TXNIP inhibition was achieved at 10 μmol/L, which was used for subsequent experiments.

To clarify TRA2A’s role in targeting TXNIP and regulating downstream pathways, THP-1 cells infected with HIV-1 Bal were treated with SRI-37330. TXNIP, NLRP3, Caspase-1, and GSDMD expression were significantly reduced (*P* < 0.05; Fig. 5C). Finally, in TRA2A knockdown THP-1 cells infected with the HIV-1 Bal virus, under the conditions of adding or not adding the TXNIP inhibitor, the expression of the NLRP3/Caspase-1/GSDMD signaling pathway protein levels was detected. The results showed that after the addition of the TXNIP inhibitor, the upregulation effect of the NLRP3/Caspase-1/GSDMD signaling pathway, which was upregulated by TRA2A knockdown, was weakened. Notably, in the absence of HIV infection, treatment with the TXNIP inhibitor reversed the upregulation of pyroptosis. However, under HIV-1 infection conditions, the regulatory role of TRA2A in macrophage pyroptosis is significantly enhanced (Fig. 5D). The ELISA detection of the downstream pro-inflammatory cytokines IL-18 and IL-1β also showed that in THP-1 cells with TRA2A knockdown, after the addition of the TXNIP inhibitor, the secretion of IL-18 and IL-1β decreased (Fig. 5E), indicating that TRA2A regulates the NLRP3/Caspase-1/GSDMD signaling pathway by targeting TXNIP.

**Fig. 6:**
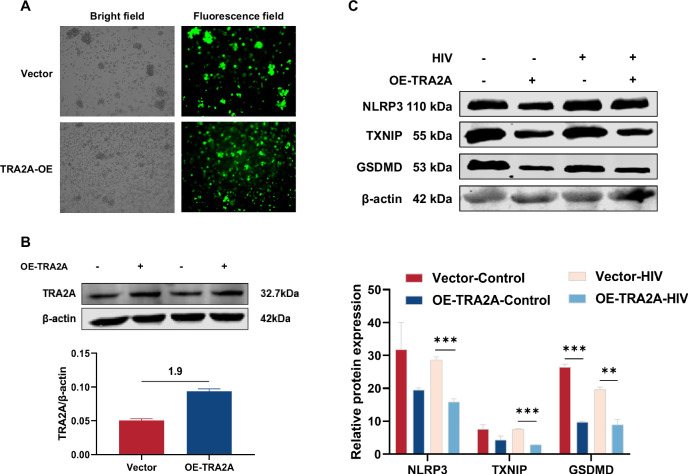
TRA2A overexpression suppresses HIV-1-induced activation of the NLRP3/Caspase-1/GSDMD pyroptosis pathway. **A** Construction of TRA2A-overexpressing macrophage cell line derived from THP-1, with confirmation of RFP fluorescence intensity; **B** WB detection of TRA2A protein expression levels in Vector and OE-TRA2A cells; **C** Overexpression of TRA2A suppressed the NLRP3/Caspase-1/GSDMD pyroptosis pathway in THP-1-derived macrophages after HIV-1 infection.

To further clarify the role that TRA2A plays in inhibiting the pyroptosis and inflammatory response of THP-1 macrophages, the overexpression of TRA2A was conducted in this study. The overexpression level of TRA2A has been verified (Fig. 6A, B), with an overexpression fold of 1.9 times. Based on this, the TRA2A overexpression strain and the control strain were respectively infected or not infected with the HIV-Bal virus. After being cultured for 24 h, the levels of downstream pathway factors were detected. It was found that the key pyroptosis and inflammatory factors in the downstream were significantly inhibited (Fig. 6C).

**Fig. 7:**
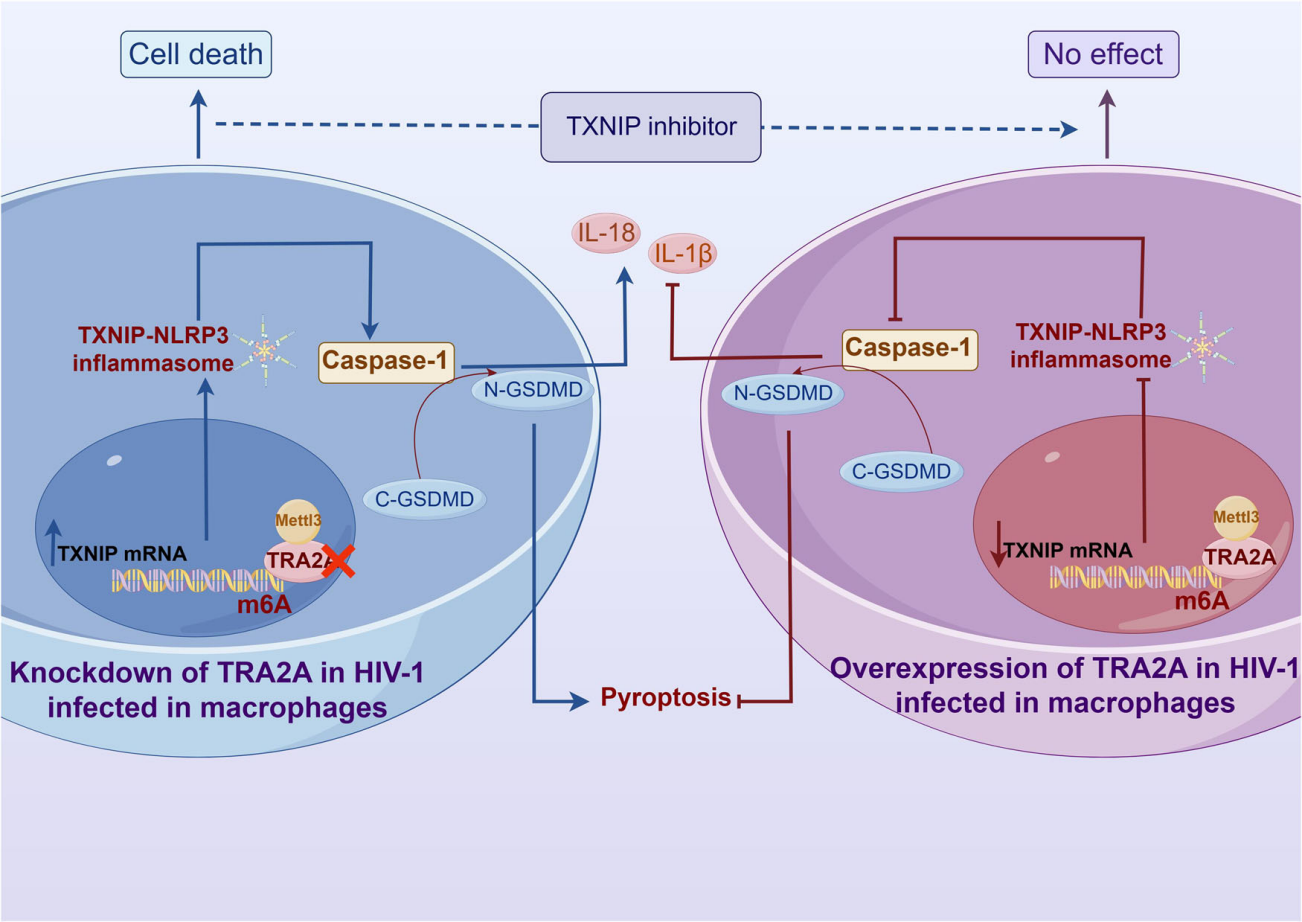
Schematic diagram of the TRA2A-mediated m6A modification regulatory axis in HIV-1-induced macrophage pyroptosis.

The above results show that knockdown of TRA2A can upregulate the TXNIP/NLRP3/Caspase-1/GSDMD signaling pathway, and the TXNIP inhibitor can downregulate the NLRP3/Caspase-1/GSDMD signaling pathway that was upregulated due to TRA2A knockdown, indicating that TRA2A regulates the NLRP3/Caspase-1/GSDMD signaling pathway by targeting the expression of TXNIP. (Fig. 7).

## Discussion

This study systematically investigated the role and underlying mechanisms of the m6A regulatory factor TRA2A in HIV-1 infection using MeRIP-seq, q-PCR, protein expression analysis, and cellular models. The results demonstrated a significant downregulation of TRA2A expression in macrophages infected with HIV-1. Knockdown of TRA2A promoted viral replication, accompanied by the activation of the TXNIP/NLRP3/Caspase-1/GSDMD signaling pathway and the release of inflammatory cytokines. Further experiments using a TXNIP inhibitor and TRA2A overexpression confirmed that TRA2A exerts its antiviral and anti-inflammatory effects by regulating TXNIP, thereby inhibiting pyroptosis and inflammatory responses. These findings highlight the important antiviral and anti-inflammatory functions of TRA2A in HIV-1 infection.

In this study, we identifies a novel mechanism by which TRA2A restricts HIV-1 replication through m6A-mediated regulation of TXNIP and subsequent modulation of the NLRP3 inflammasome-pyroptosis axis. Our findings demonstrate that TRA2A deficiency in HIV-1-infected macrophages leads to increased stability and expression of TXNIP mRNA, which directly activates the NLRP3 inflammasome complex [17]. Activated NLRP3 promotes Caspase-1-dependent cleavage of GSDMD, triggering pyroptosis [18]—a lytic cell death pathway that paradoxically enhances viral dissemination by releasing infectious particles and pro-inflammatory cytokines. Importantly, TRA2A overexpression or pharmacological inhibition of TXNIP reversed this cascade, restoring control over both viral replication and inflammation. These observations align with emerging evidence that m6A modifications fine-tune innate immune responses by destabilizing pro-inflammatory transcripts, such as TXNIP, which has been implicated in amplifying inflammasome signaling across viral infections. The dual role of TRA2A—suppressing viral propagation while mitigating immunopathology—highlights its potential as a therapeutic target to disrupt the vicious cycle of HIV-1-induced inflammation and reservoir persistence [19].

M6A is the most prevalent RNA modification in eukaryotes and can indirectly influence viral replication by modulating the expression of RNAs involved in viral processes [20]. Potential m6A modification sites on TXNIP were predicted using the SRAMP software, and the site with the highest confidence was selected for further analysis. A SELECT assay primer was designed to evaluate the m6A modification abundance at this site. The results revealed a significant reduction in m6A modification abundance at the selected site in TRA2A-knockdown cells compared to controls. Subsequently, RIP assays confirmed the direct interaction between TRA2A and TXNIP, identifying a specific m6A modification site on TXNIP regulated by TRA2A. This modification site was found to influence the NLRP3/Caspase-1/GSDMD signaling pathway, thereby affecting the secretion of inflammatory cytokines IL-1β and IL-18. These findings highlight a novel regulatory mechanism by which TRA2A modulates inflammation through m6A-dependent pathways. TRA2A may limit the decisive steps of HIV-1 post-integration LTR-driven transcription/translation by suppressing TXNIP/NLRP3 inflammasome-driven pyroptosis and inflammation. The NLRP3 inflammasome is a central mediator of HIV-1-related inflammatory signals, and regulating its activity can interrupt HIV-1 replication and transcription [21].

Clinically, TRA2A offers a promising host-directed therapeutic target for ameliorating HIV-associated inflammation. While ART has been shown to improve certain immunological parameters, such as increasing CD4^+^ T-cell counts and reducing inflammation [22], its impact on pro-inflammatory cytokines, particularly IL-1β, remains controversial [23, 24]. In addition, long-term ART may lead to multi-organ systemic complications such as chronic inflammation [25] and metabolic disorders [26, 27], with 30% of HIV-infected individuals having incomplete immune reconstitution on ART [28], which is associated with persistent inflammatory responses and disease progression. Stimulation of the TRA2A pathway on NLRP3/Caspase-1/GSDMD can reduce Caspase-1 activation and pyroptosis levels, thereby protecting macrophages from death and promoting the survival and functional recovery of immune cells, This helps alleviate immunodeficiency and improve immune reconstitution following antiviral therapy [29]. Furthermore, the activation of the NLRP3 inflammasome drives HIV-1-associated inflammatory signals, leading to systemic inflammation and various comorbidities [30]. Inhibiting the TXNIP/NLRP3 interaction can downregulate inflammasome activity and reduce the production of pro-inflammatory factors, thereby controlling the inflammatory cascade. This helps reduce the inflammatory burden and the risk of complications associated with HIV-1 infection [31]. Pyroptosis, an inflammasome-dependent form of programmed cell death, is not restricted to HIV-1 infection but a conserved defensive feature ubiquitous in hosts challenged by viruses such as influenza A virus [32] and hepatitis B/C viruses [33, 34], as well as bacteria including *Mycobacterium tuberculosis* [35] and *Salmonella spp* [36]. Aberrant activation of the TXNIP-NLRP3 pathway plays a central role in the immunopathological damage of various infectious diseases and may serve as a common molecular mechanism driving excessive inflammatory responses following infections by different pathogens. Future investigations should extend to coinfection settings to elucidate the functional heterogeneity of TRA2A in infections with different pathogens, which may lay the groundwork for the development of broad-spectrum regulatory targets against immunopathological injury in infectious diseases.

There are some limitations in this study. First, in this study, we have demonstrated that TRA2A affects HIV-1 replication by regulating the levels of macrophage pyroptosis and inflammatory response, as evidenced by changes in P24 levels, but has not yet been individually validated for each step of the HIV-1 life cycle. Second, this research focused on macrophage models to explore the role of TRA2A in regulating HIV-1 replication and pyroptosis. The relevance of TRA2A in other cell types involved in HIV-1 infection remains unexplored and warrants further investigation.

## Conclusions

Our current study elucidates the role and underlying mechanisms of the m6A regulatory factor TRA2A in HIV-1 infection. The findings demonstrate that TRA2A expression is significantly downregulated in individuals with progressive HIV-1 infection, particularly in macrophages. Subsequent cellular experiments confirmed that TRA2A suppresses HIV-1 replication in macrophages and regulates TXNIP expression by modulating its m6A modifications. This regulation impacts the NLRP3/Caspase-1/GSDMD signaling pathway, thereby suppresses pyroptosis and inflammatory responses. These findings provide novel insights into the pathogenesis of HIV-1 infection and identify TRA2A as a potential molecular target for the development of innovative therapeutic strategies.

## Materials and methods

### Clinical sample collection

Participants were divided into two groups: healthy controls and individuals with typical HIV-1 progression. The inclusion criteria for the TPs were as follows: (1) age ≥18 years, with a confirmed diagnosis of HIV-1 infection; (2) HIV-1 infection had not progressed to AIDS, and no characteristic symptoms of AIDS were present during clinical evaluation. HIV-1 diagnosis adhered to the “Diagnostic Criteria for AIDS and HIV Infection” (WS293-2019) outlined in the health industry standards of the People’s Republic of China; (3) HIV-1 positive confirmed 3–9 years prior to inclusion, without antiretroviral therapy, and CD4^+^ T lymphocyte counts progressively decreased to <500 cells/µL. Exclusion criteria included the presence of other opportunistic infections or infectious diseases, malignancies, chronic diseases, or psychiatric disorders. The inclusion criteria for healthy controls were: no history of disease, no medication use in the past week, no abnormal symptoms such as fever. This study adhered to informed consent principles, recruit individuals with TPs who meet the inclusion criteria in the Guangxi region, and match HCs locally.

### PBMCs isolation and culture section

Peripheral blood (10 mL) was collected into EDTA-coated tubes and processed using GE Ficoll-Paque PLUS (GE Healthcare, USA) and SepMate tubes (Stemcell Technologies, USA). Blood was centrifuged at 2500 rpm for 10 min to separate plasma, followed by dilution with PBS containing 2% fetal bovine serum (FBS, Gibco, USA) and layering over Ficoll-Paque. After centrifugation at 1200 g for 10 min, the mononuclear cell layer was transferred to a new tube, washed with PBS, and centrifuged at 300 g for 10 min. Red blood cell lysis was performed if necessary, followed by a final wash. The PBMC pellet was resuspended in complete DMEM (Gibco, USA) containing 10% FBS and antibiotics and cultured with GM-CSF for 5-7 days to differentiate into macrophages.

### Cell culture

The THP-1 cell line used in this study was purchased from the Cell Bank of the Shanghai Institute of Biological Sciences, Chinese Academy of Sciences. The cells were purchased in 2018 and confirmed to be free of contamination. The THP-1 cell line is a human monocytic cell line, cultured in RPMI 1640 medium (Gibco, USA), supplemented with 10% FBS (Gibco, USA), and 1% penicillin/streptomycin mixture (Gibco, USA) under standard conditions of 37 °C and 5% CO₂. To differentiate THP-1 cells into macrophage-like cells, cells were seeded into 6-well plates and treated with 100 nM of Phorbol 12-myristate 13-acetate (PMA, Sigma, USA) for 24 h. After differentiation, these cells were used for subsequent HIV-1 infection experiments.

### Real-time qPCR

Total RNA was reverse transcribed using the TaKaRa RR036A One Step SYBR PrimeScript RT-PCR Kit (Perfect Real Time) following the manufacturer’s protocol. qRT-PCR was performed on a LightCycler 480 Real-Time PCR System (Roche) with SYBR Green detection. Target gene expression levels were normalized to GAPDH using the 2^(-ΔΔCt) method.

### Western blot

Western blotting was conducted with the Bio-Rad Western Blot Electrophoresis and Transfer System (Bio-Rad Laboratories, Hercules, CA, USA). Total cellular proteins were extracted via RIPA lysis buffer containing 1% PMSF and quantified by the BCA assay. Equal amounts of protein (20–50 μg per lane) were separated by 10%–12% SDS-PAGE, then electrotransferred onto PVDF membranes (Merck, USA) at 250 mA for 90 min. Membranes were blocked with 5% non-fat milk in TBST for 2 h, incubated overnight at 4 °C with primary antibodies, and then for 1 h with secondary antibodies—all antibodies were purchased from Cell Signaling Technology (CST, USA). Protein bands were visualized by ECL and quantified using Image Studio software, with β-Actin as the internal reference.

### ELISA

HIV-1 p24 antigen levels were measured using ELISA kits provided by the Biomedical Engineering Center of Hebei Medical University, following the manufacturer’s instructions. Cytokine levels of IL-18 and IL-1β were quantified using ELISA kits obtained from Quanzhou Ruixin Biotechnology Co., Ltd. All samples were analyzed in triplicate, and absorbance was measured at 450 nm using a microplate reader (Thermo Fisher Scientific). Cytokine concentrations were calculated based on standard curves generated for each assay.

### SELECT qPCR detection of m6A modification site differences

M6A modification site differences were detected via Epi-SELECT™ kit (Guangzhou Epigenetic Biotech) and SELECT qPCR: total RNA extracted, reverse-transcribed to cDNA, quantified with specific primers, assessed group differences per protocol via qPCR.

### RNA immunoprecipitation (RIP) assays

RIP assays were performed using a kit from Guangzhou Boshin Biotechnology, strictly following the manufacturer’s protocols. Briefly, 20 million cells were collected, washed twice with PBS, and lysed in lysis buffer supplemented with protease inhibitors and RNase inhibitors to prevent degradation. After DNase treatment to remove genomic DNA, Protein A/G beads were equilibrated with lysis buffer. Cell lysates were divided into IP, IgG, and Input fractions; the IP and IgG fractions were incubated with specific TRA2A antibody (CST, USA) at 4°C for 16 h on a vertical shaker. Immune complexes were captured by Protein A/G beads, separated magnetically, and thoroughly washed. Finally, RNA was extracted using phenol-chloroform-isoamyl alcohol, quantified by NanoDrop, reverse-transcribed, and analyzed by nucleic acid electrophoresis and RT-qPCR to verify RNA-protein interactions.

### Transmission electron microscopy (TEM)

Cell pellets were harvested by centrifugation and fixed with EM fixative at 4 °C for 2-4 h, followed by pre‑embedding with 1% agarose. Samples were then post‑fixed with 1% osmium tetroxide, dehydrated in graded ethanol and acetone, infiltrated and embedded in 812 resin, and polymerized at 60 °C for 48 h. After 60–80 nm ultrathin sectioning and staining with uranyl acetate and lead citrate, ultrastructural observation was performed using Hitachi HT7800/HT7700 transmission electron microscope.

### Data analysis

Data preprocessing was performed using R Studio software (version 3.6.1; R Studio, Boston, MA, USA). Statistical analyses were conducted using SPSS software (version 23.0). Categorical variables were compared using the χ² test or Fisher’s exact test. Experimental data were expressed as mean ± standard deviation or relative fold changes, and comparisons were made using t-tests or analysis of variance (ANOVA). GraphPad Prism software (version 9.5) was used to generate graphical representations of the results. A significance level of α = 0.05 was set for all statistical tests, and two-tailed tests were applied. Results with *P* < 0.05 were considered statistically significant.

## Supplementary information

Supplementary Table 1

Original Data Files

Supplementary Figure 1

Supplementary Figure 2

Table S2. Demographic characteristics of the study population after matching [n (%)]

## Data Availability

The datasets generated and/or analyzed during the current study are not publicly available due to restrictions imposed by the ethical review board to protect participant confidentiality. However, they are available from the corresponding author upon reasonable request and with appropriate institutional approvals.
