# Supplementary material for: TRA2A negatively regulates HIV-1-induced macrophage pyroptosis by mediating TXNIP expression in an m6A-dependent manner
Source: Cell Death Discov. 2026 Jun 26;12:282. doi: 10.1038/s41420-026-03236-2 (PMC13309537; doi:10.1038/s41420-026-03236-2)
Supplement: Supplementary file 1 — Supplementary Table 1 [file 41420_2026_3236_MOESM1_ESM.docx]

| **Table S1. Demographic characteristics of the study population [n (%)]** | | | | |
| --- | --- | --- | --- | --- |
| **Variable** |  | **Healthy**  **(N=60)(n,%)** | **TP**  **(N=54)(n,%)** | ***P*** |
| **Age(years)** |  | 29±3 | 38±2 | <0.001 |
| **Gender** |  |  |  | <0.001 |
|  | Man | 35(58.3) | 49(90.7) |  |
|  | Female | 25(41.7) | 5(9.3) |  |
| **Nationality** |  |  |  | 0.01 |
|  | Han | 47(78.3) | 29(53.7) |  |
|  | Zhuang | 13(21.7) | 22(40.7) |  |
|  | Other | 0(0.0) | 3(5.6) |  |
| **Marital status** |  |  |  | 0.082 |
| Single, divorced, widowed | | 43(71.7) | 33(61.1) |  |
|  | Married | 17(28.3) | 17(31.5) |  |
|  | Other | 0(0.0) | 4(7.4) |  |
| **BMI (kg/m^2^)** |  |  |  | <0.001 |
|  | <18.5 | 9(15.0) | 35(64.8) |  |
|  | 18.5-24 | 38(63.3) | 6(11.1) |  |
|  | 24.1-30 | 13(21.7) | 11(20.4) |  |
|  | >30 | 0(0.0) | 2(3.7) |  |
