# Supplementary material for: TRA2A negatively regulates HIV-1-induced macrophage pyroptosis by mediating TXNIP expression in an m6A-dependent manner
Source: Cell Death Discov. 2026 Jun 26;12:282. doi: 10.1038/s41420-026-03236-2 (PMC13309537; doi:10.1038/s41420-026-03236-2)

Figure 1D

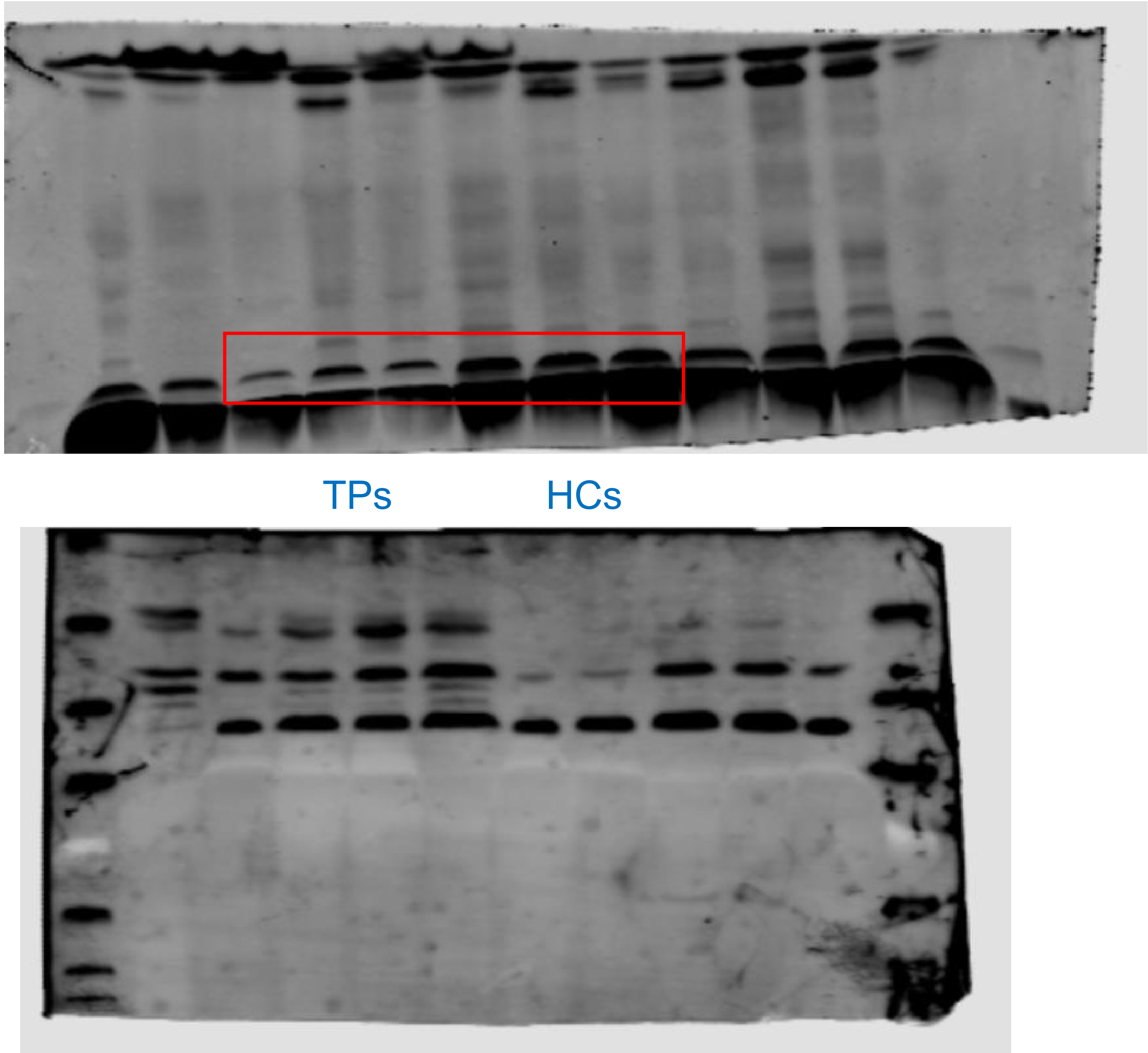

Ab：TRA2A

Ab：GAPDH

Figure 1F

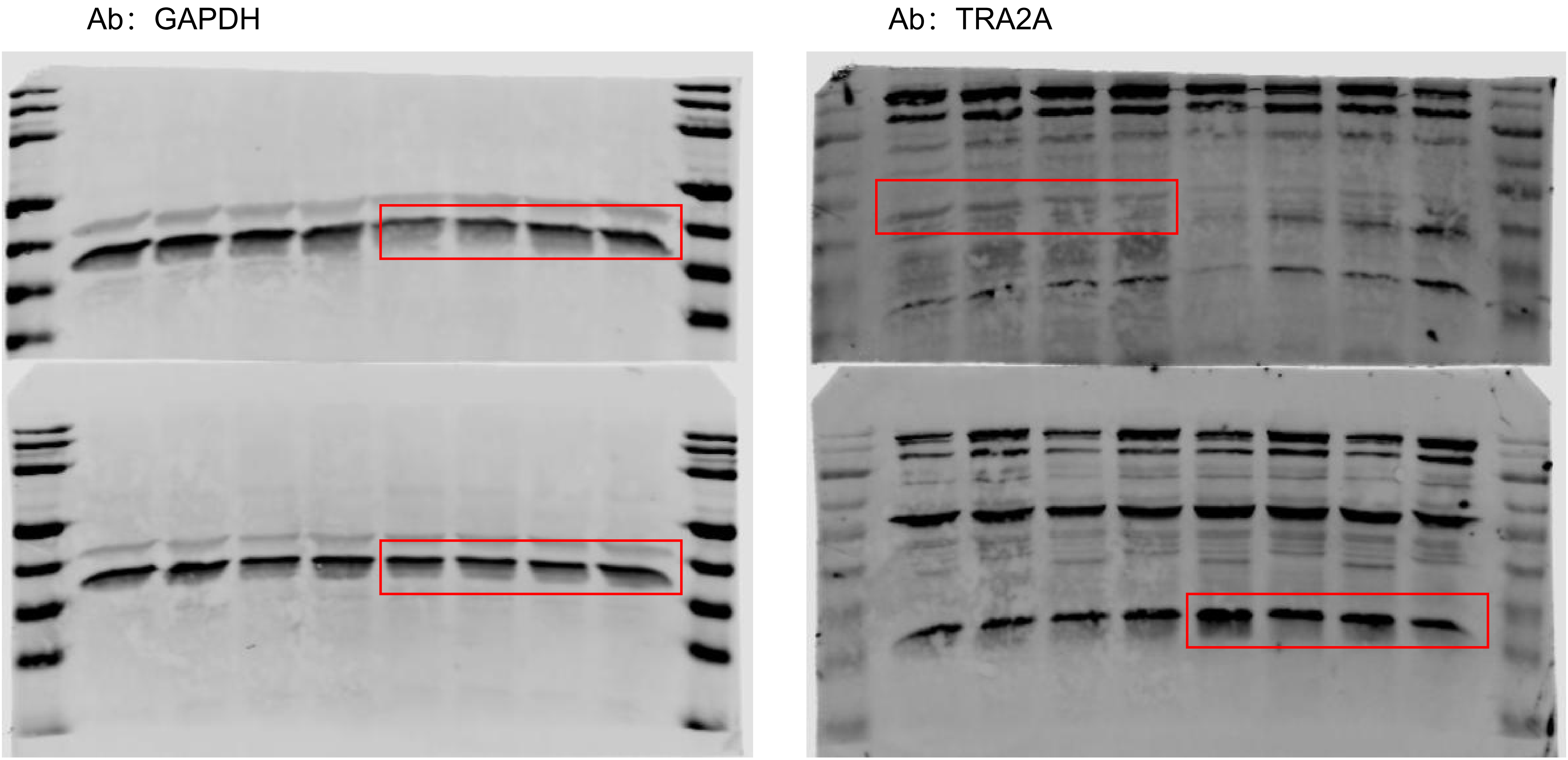

Figure 2D

Ab：β-actin

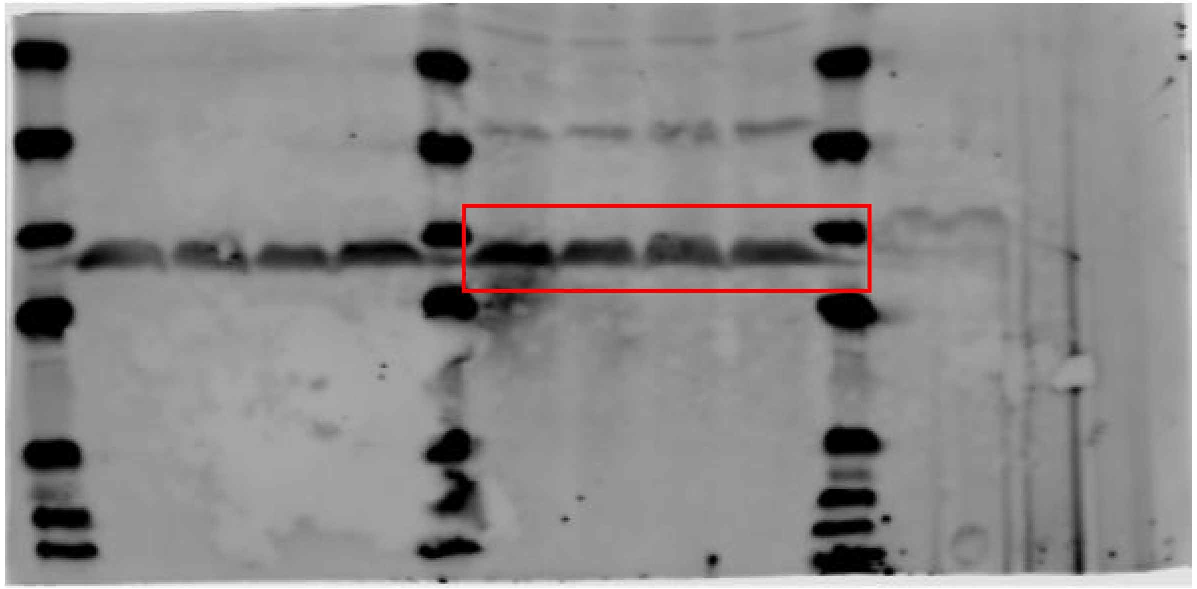

Ab：TRA2A

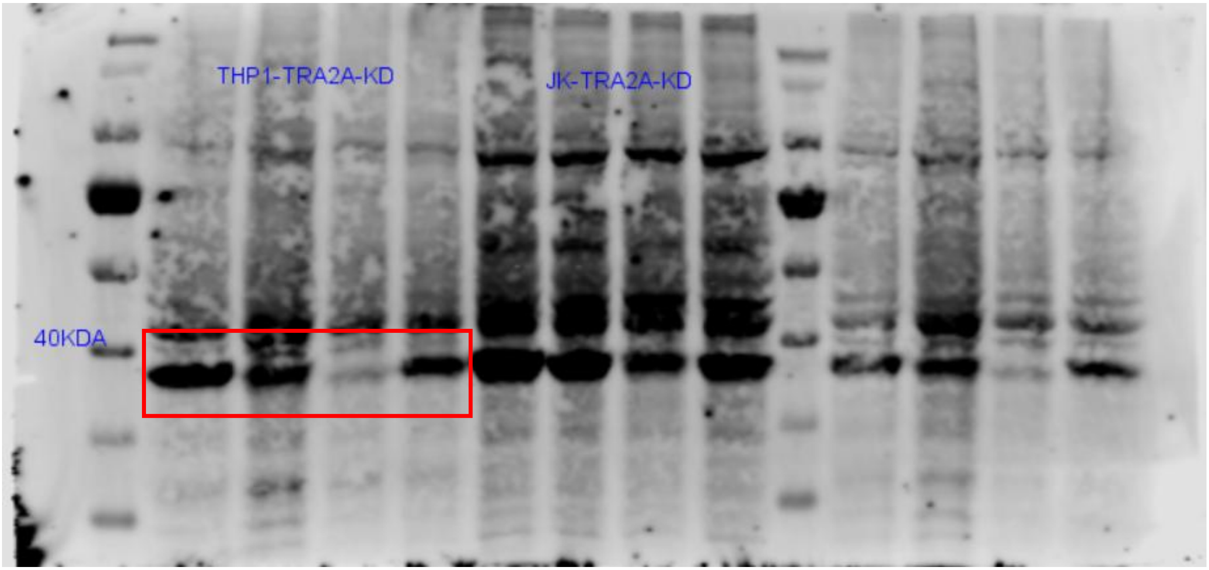

Figure 3F

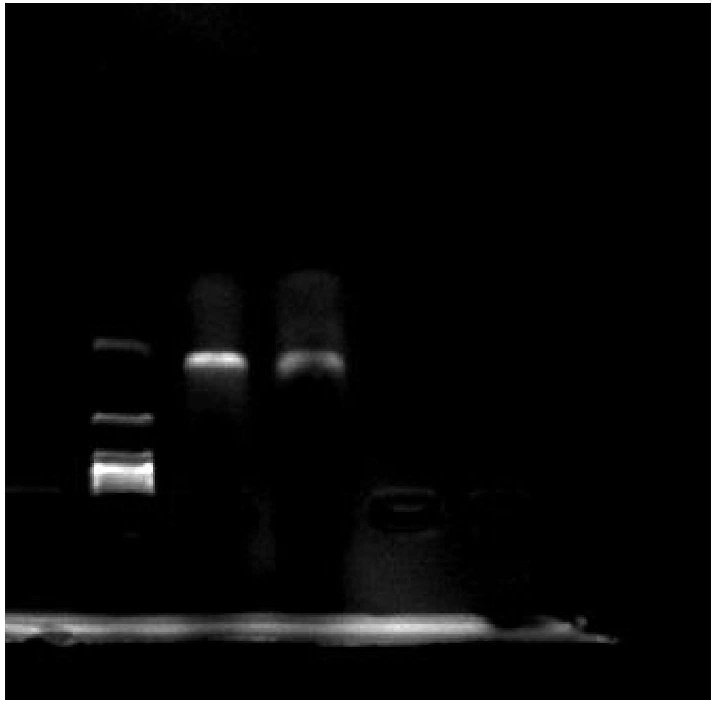

Figure 4C

Ab： NLRP3

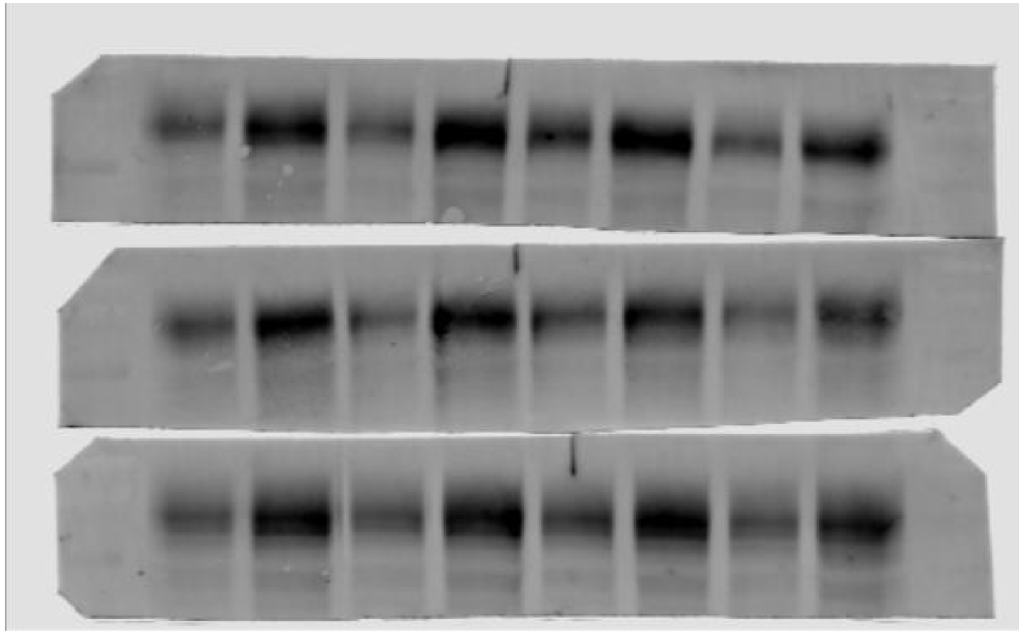

Ab： GSDMD

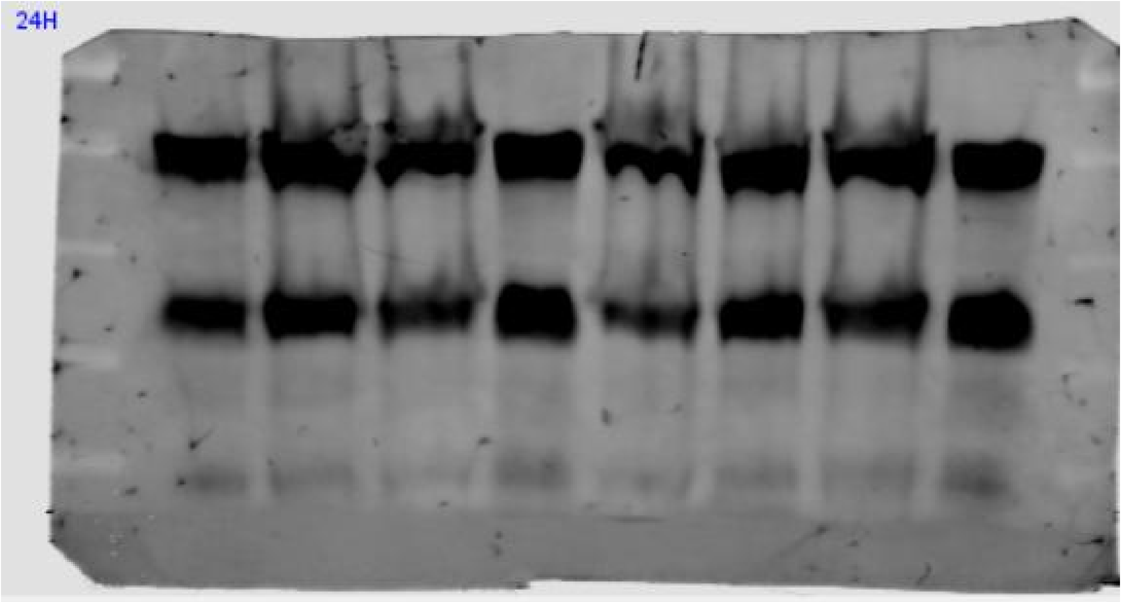

Ab： TXNIP

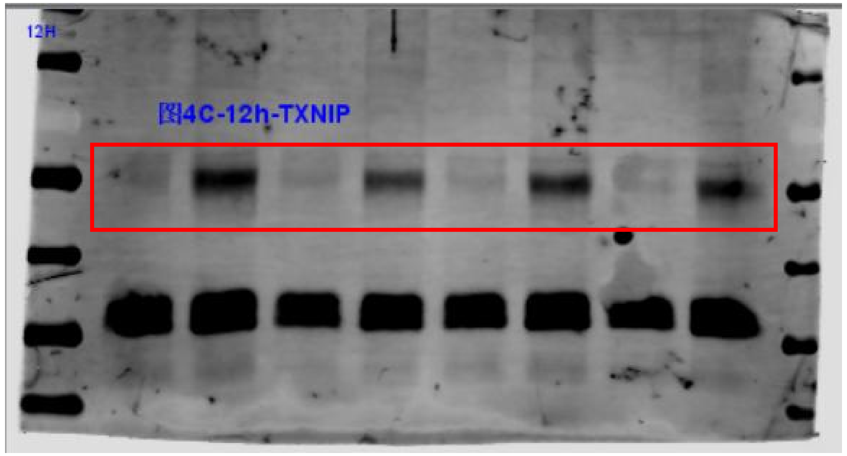

Figure 4C

Ab： β-actin

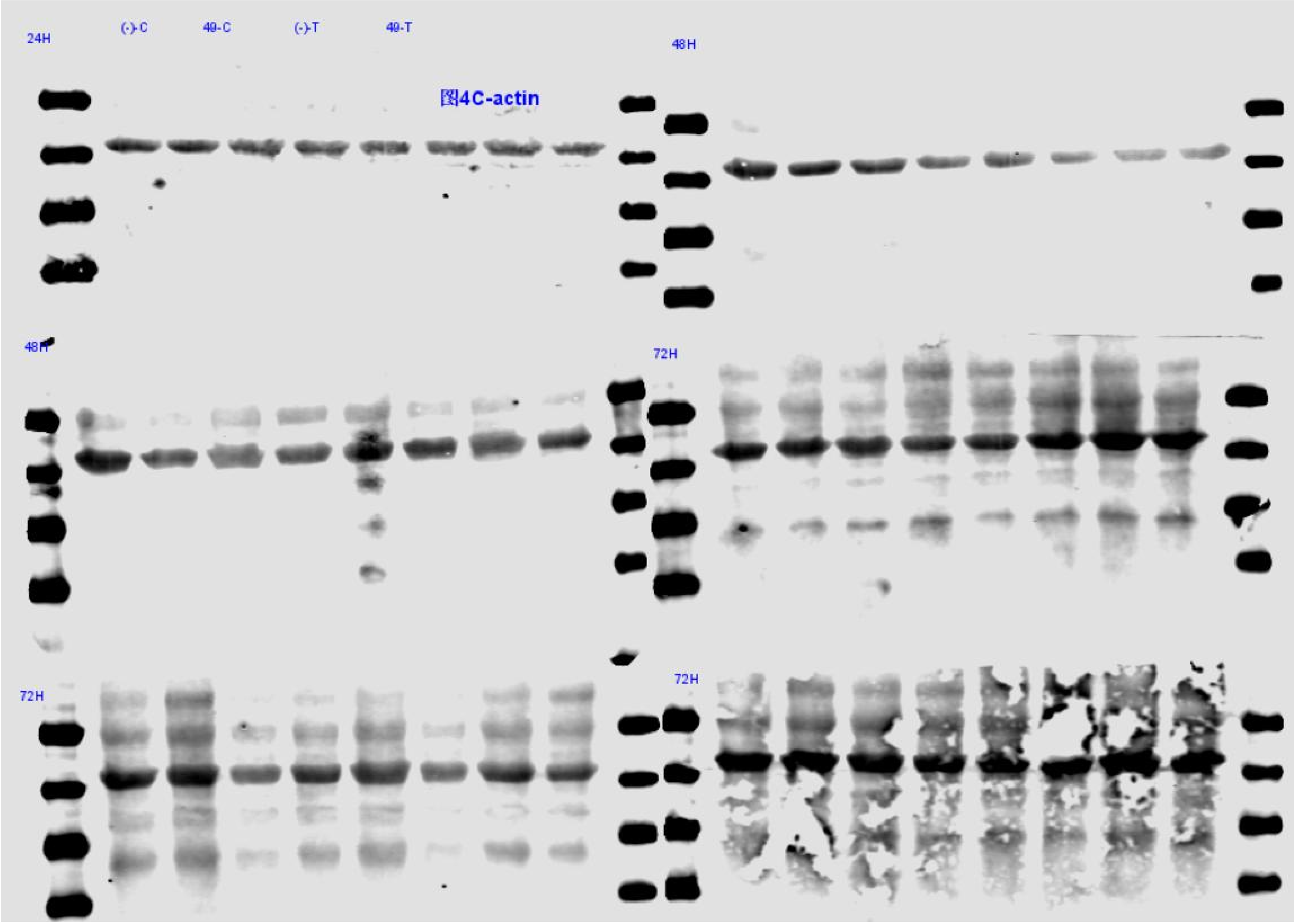

Figure 5B

Ab：β-actin

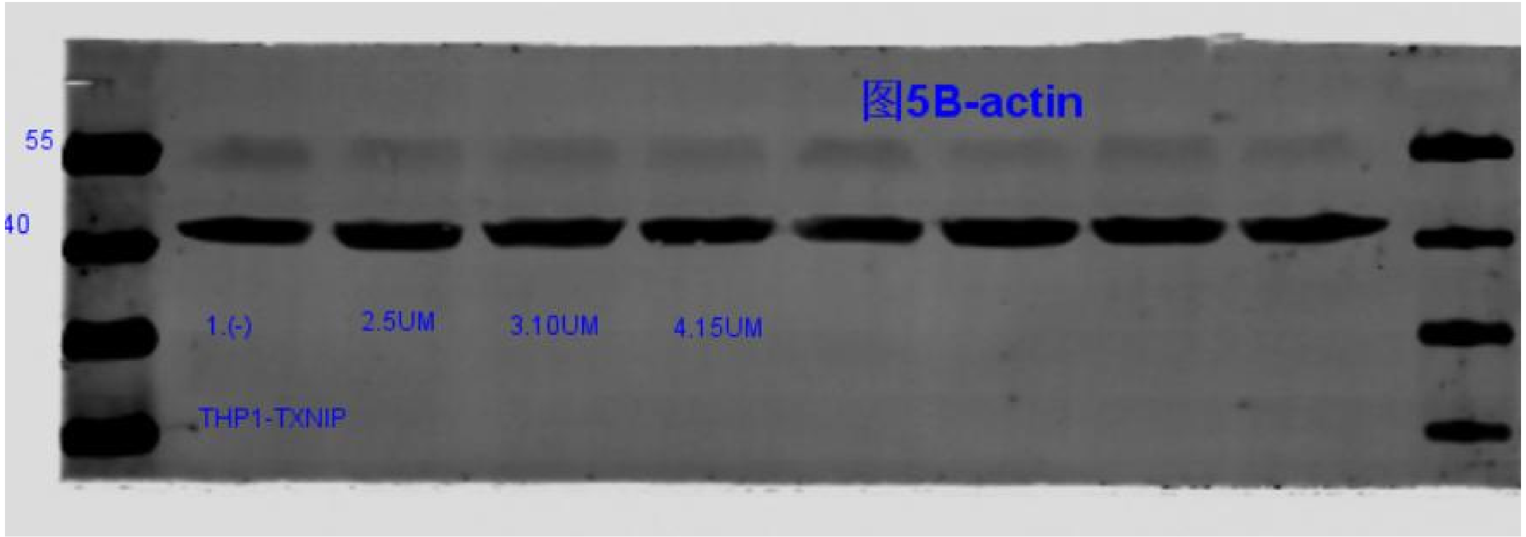

Ab：TXNIP

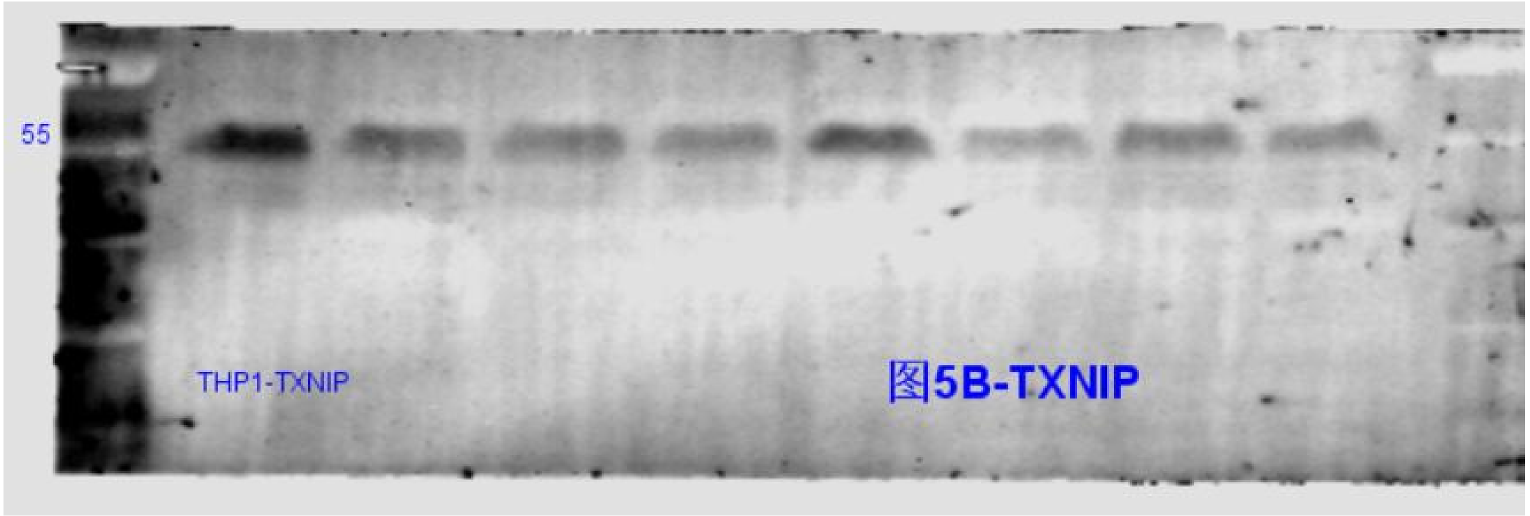

Figure 5C

Ab： caspase-1

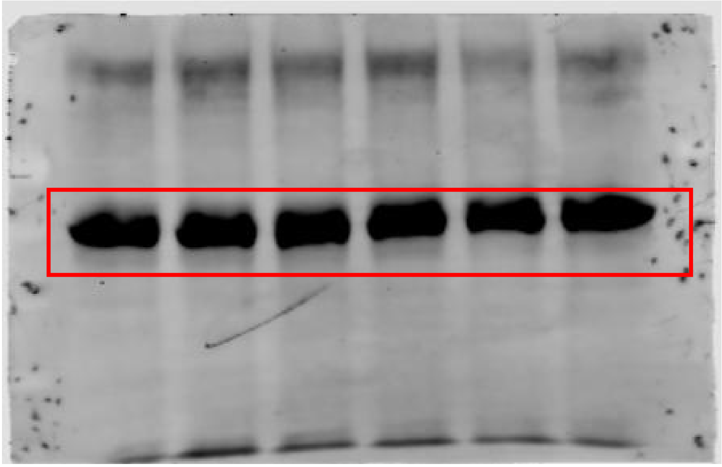

Ab： NLRP3

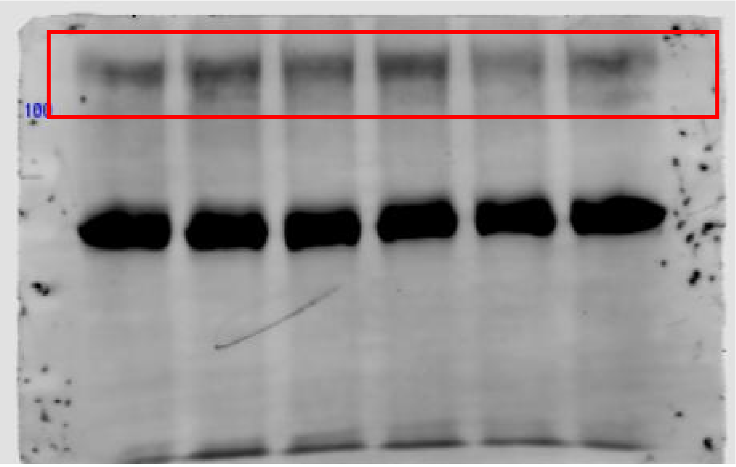

Ab： GSDMD

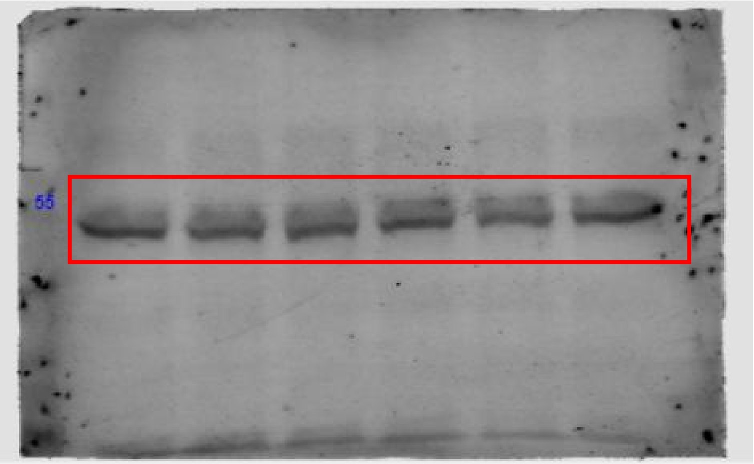

Ab： GAPDH

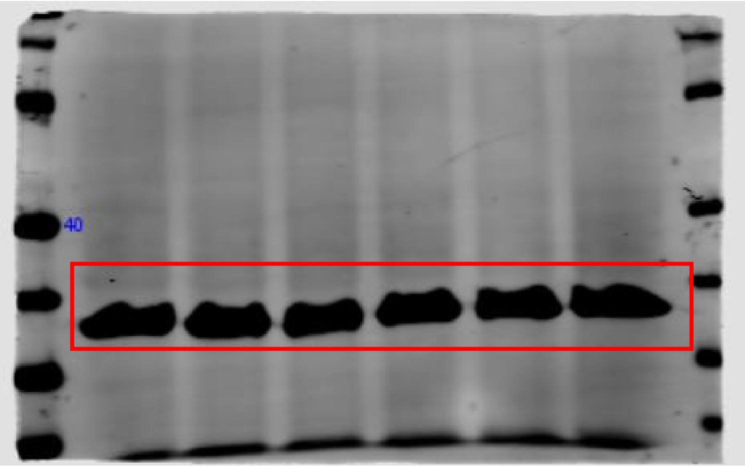

Figure 5C

Ab： caspase-1

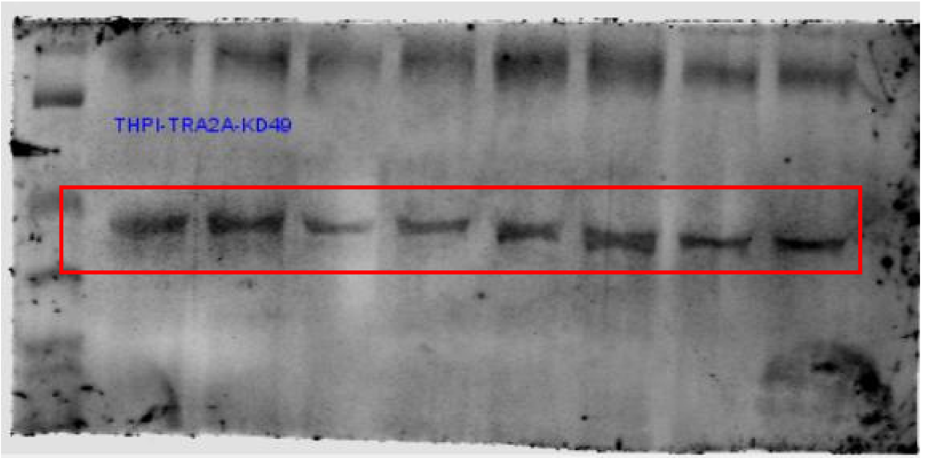

Ab： TXNIP

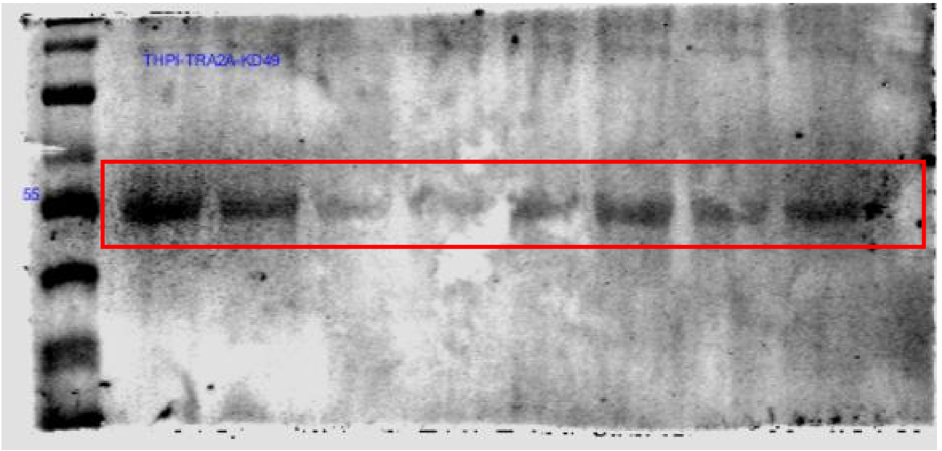

Ab： NLRP3

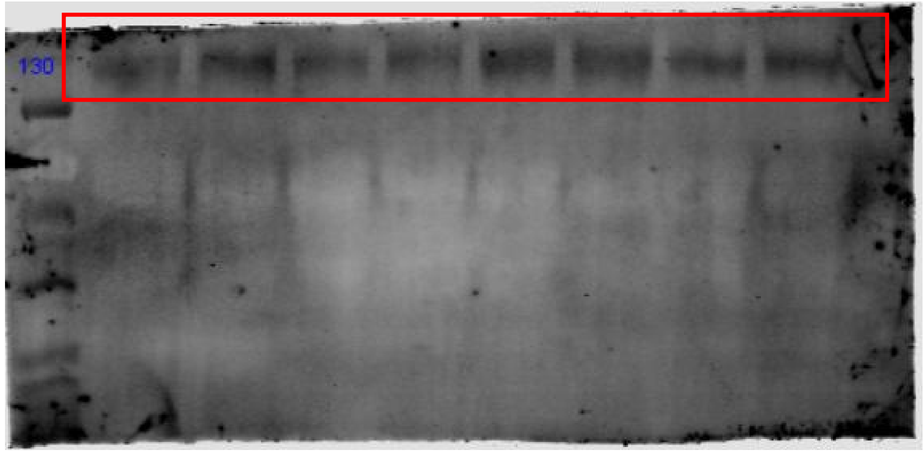

Ab： GAPDH

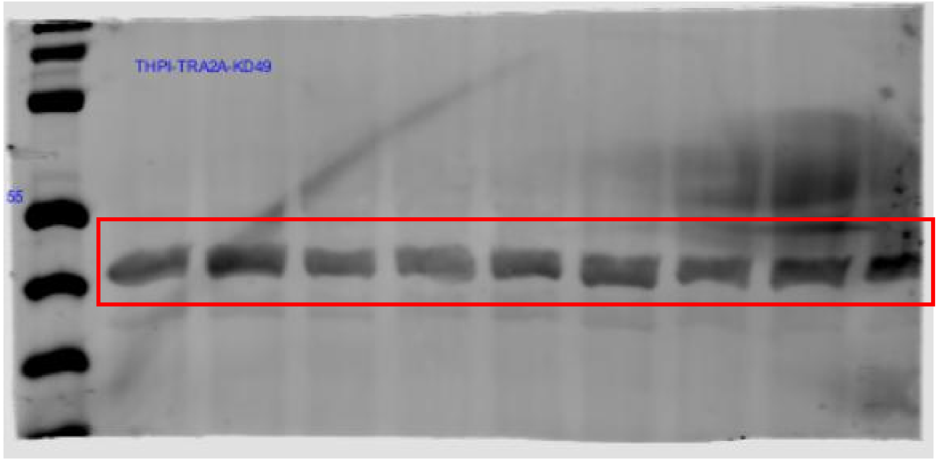

Figure 6B

Ab：TRA2A

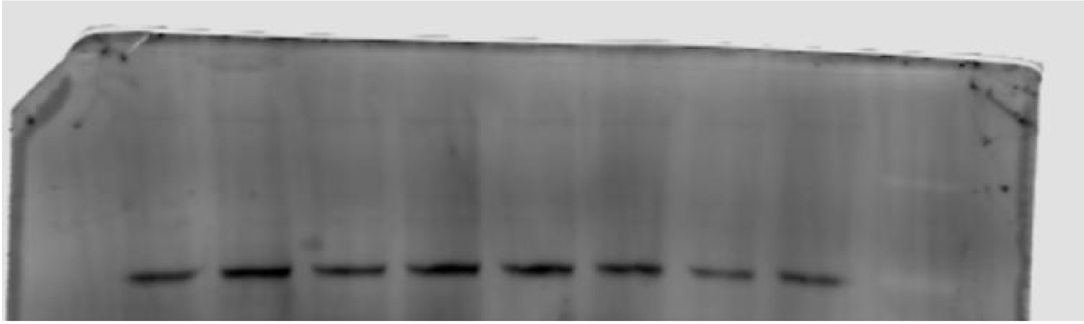

Ab：β-actin

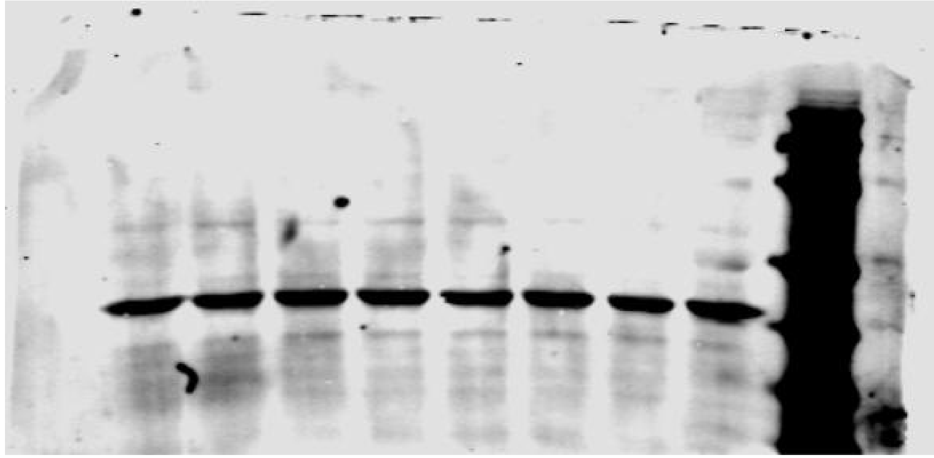

Figure 6C

Ab： β-actin

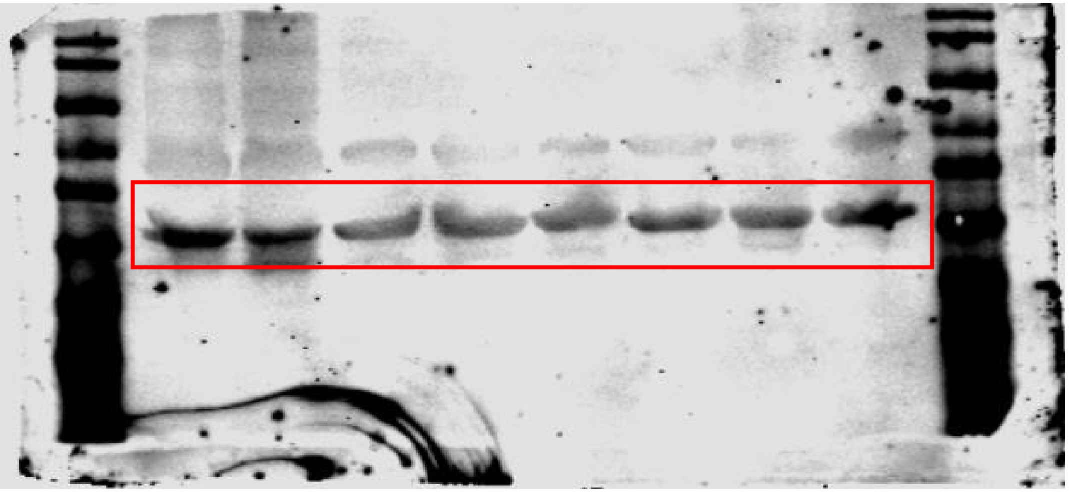

Ab： TXNIP

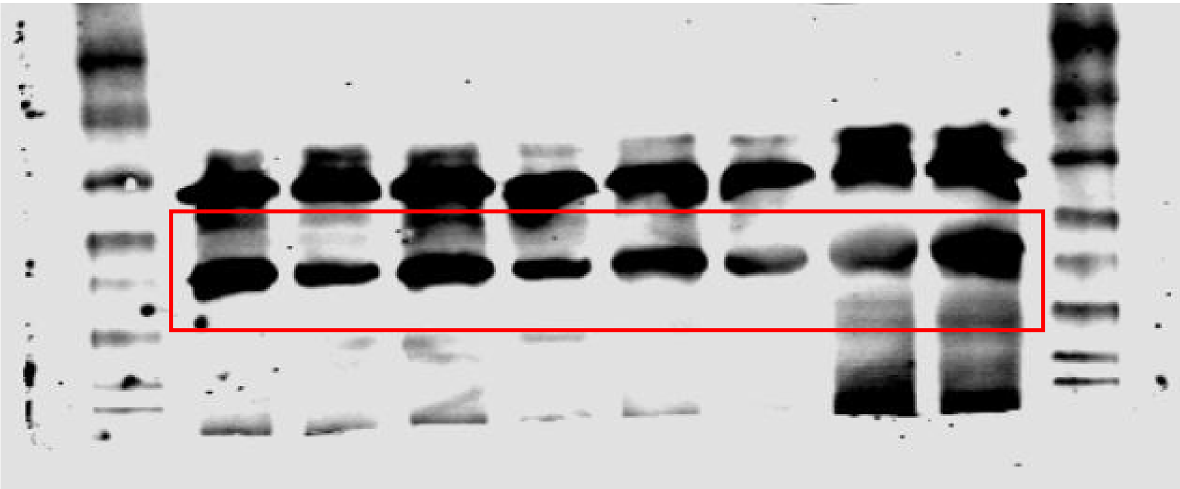

Ab： GSDMD

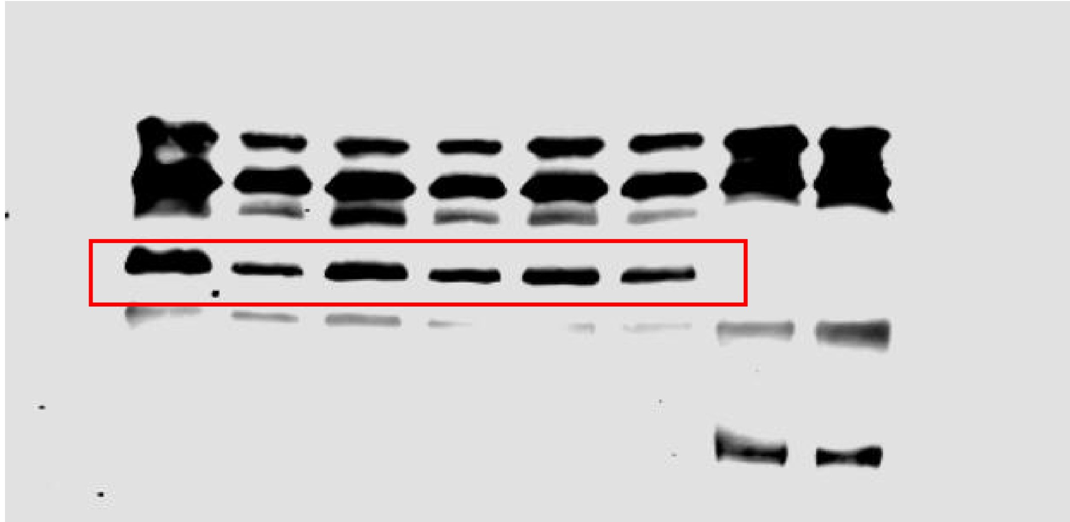

Ab： GSDMD

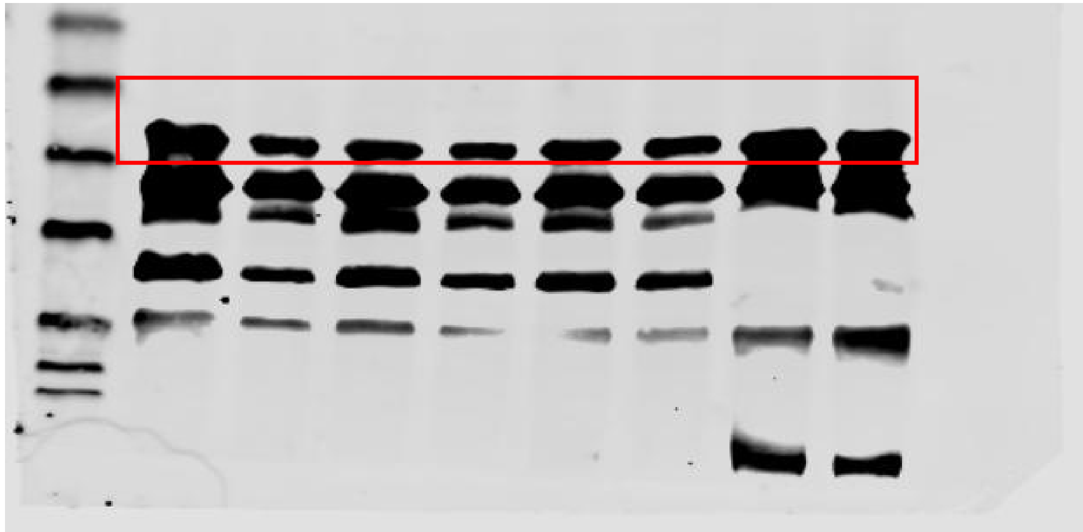

Supplement: Supplementary file 2 — Original Data Files [file 41420_2026_3236_MOESM2_ESM.pdf]
