# Supplementary material for: TRA2A negatively regulates HIV-1-induced macrophage pyroptosis by mediating TXNIP expression in an m6A-dependent manner
Source: Cell Death Discov. 2026 Jun 26;12:282. doi: 10.1038/s41420-026-03236-2 (PMC13309537; doi:10.1038/s41420-026-03236-2)
Supplement: Supplementary file 3 — Supplementary Figure 1 [file 41420_2026_3236_MOESM3_ESM.pdf]

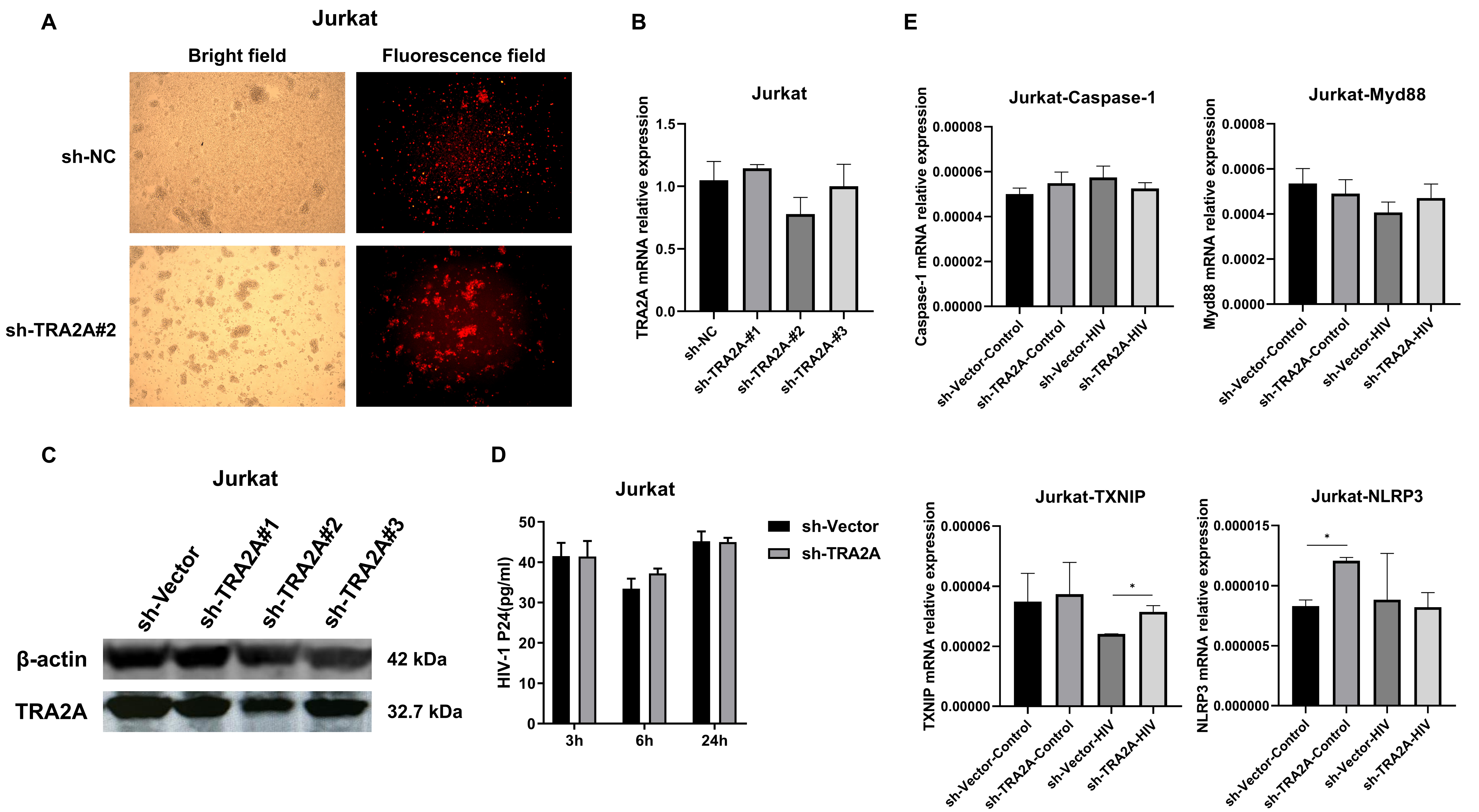

A
Jurkat
Bright field
Fluorescence field
sh-NC
sh-TRA2A#2
B
Jurkat
TRA2A mRNA relative expression
1.5
1.0
0.5
0.0
sh-NC
sh-TRA2A-#1
sh-TRA2A-#2
sh-TRA2A-#3
E
Jurkat-Caspase-1
Caspase-1 mRNA relative expression
0.00008
0.00006
0.00004
0.00002
0.00000
sh-Vector-Control
sh-TRA2A-Control
sh-Vector-HIV
sh-TRA2A-HIV
Jurkat-Myd88
Myd88 mRNA relative expression
0.0008
0.0006
0.0004
0.0002
0.0000
C
Jurkat
sh-Vector
sh-TRA2A#1
sh-TRA2A#2
sh-TRA2A#3
β-actin
42 kDa
TRA2A
32.7 kDa
D
Jurkat
HIV-1 P24(pg/ml)
50
40
30
20
10
0
3h
6h
24h
sh-Vector
sh-TRA2A
Jurkat-TXNIP
TXNIP mRNA relative expression
0.00006
0.00004
0.00002
0.00000
*
Jurkat-NLRP3
NLRP3 mRNA relative expression
0.000015
0.000010
0.000005
0.000000
*
