# Supplementary figures and images for: TRA2A negatively regulates HIV-1-induced macrophage pyroptosis by mediating TXNIP expression in an m6A-dependent manner
Source: Cell Death Discov. 2026 Jun 26;12:282. doi: 10.1038/s41420-026-03236-2 (PMC13309537; doi:10.1038/s41420-026-03236-2)

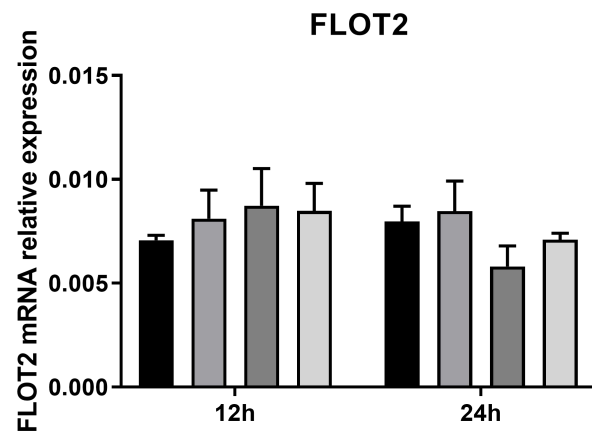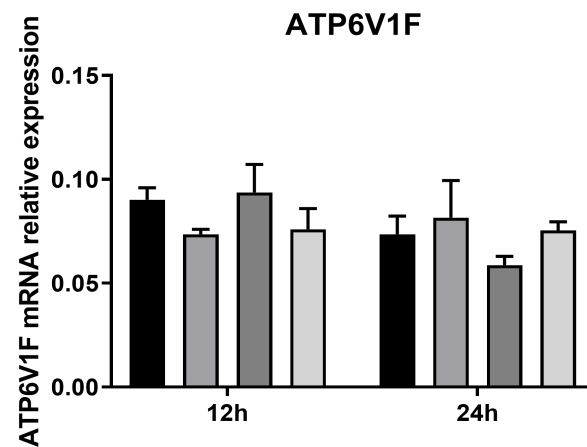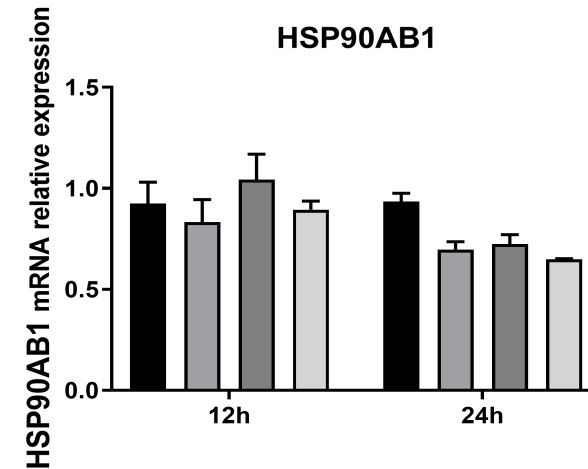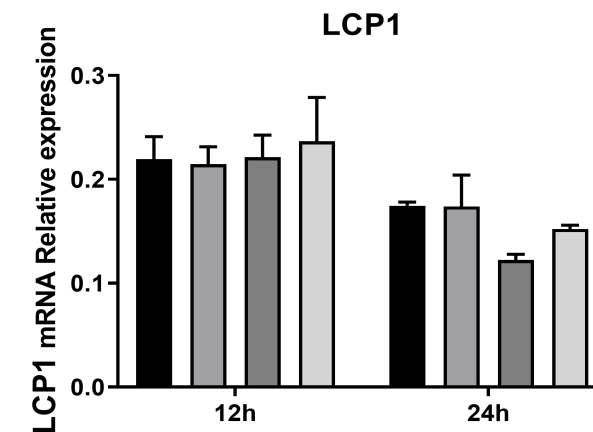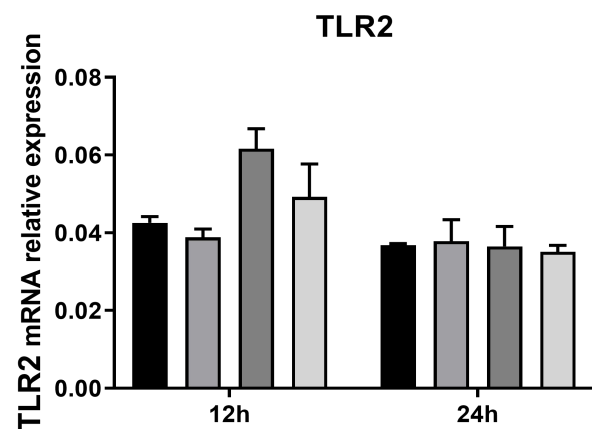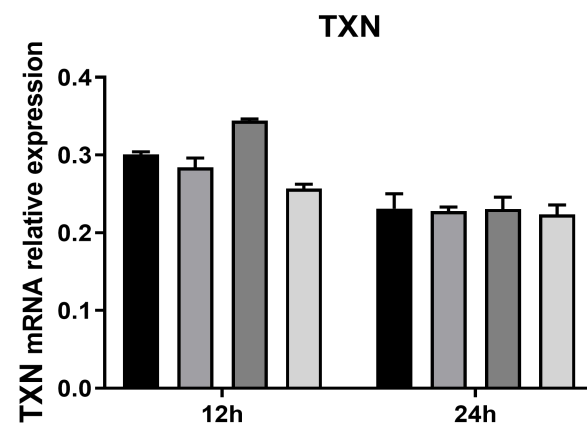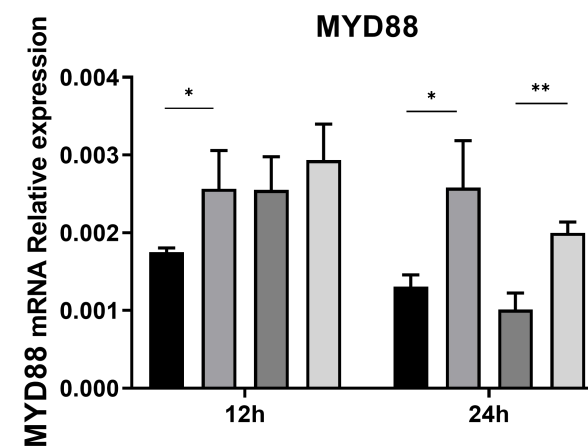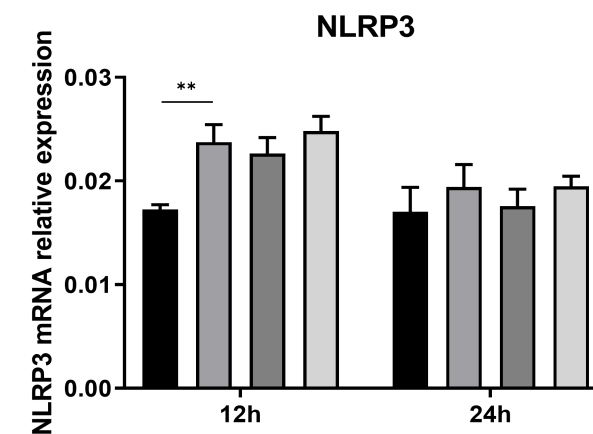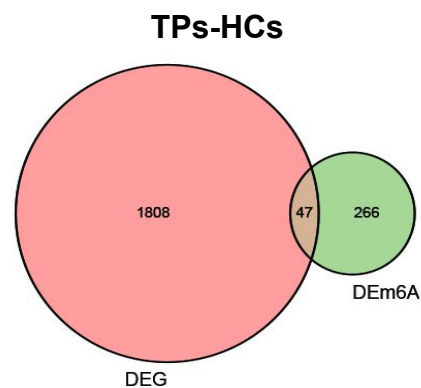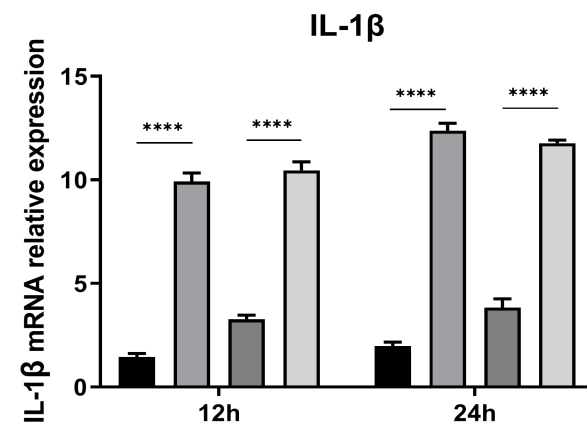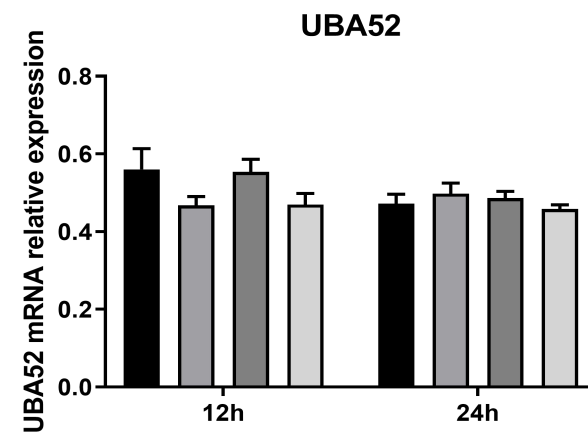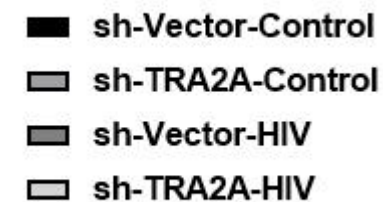

Supplement: Supplementary file 4 — Supplementary Figure 2 [file 41420_2026_3236_MOESM4_ESM.pdf]
