# Supplementary material for: TRA2A negatively regulates HIV-1-induced macrophage pyroptosis by mediating TXNIP expression in an m6A-dependent manner
Source: Cell Death Discov. 2026 Jun 26;12:282. doi: 10.1038/s41420-026-03236-2 (PMC13309537; doi:10.1038/s41420-026-03236-2)
Supplement: Supplementary file 5 — Table S2. Demographic characteristics of the study population after matching [n (%)] [file 41420_2026_3236_MOESM5_ESM.docx]

| **Table S2. Demographic characteristics of the study population after matching [n (%)]** | | | | |
| --- | --- | --- | --- | --- |
| **Variable** |  | **Healthy**  **(N=8)(n,%)** | **TP**  **(N=8)(n,%)** | ***P*** |
| **Age(years)** |  | 36±2 | 37±2 | 0.954 |
| **Gender** |  |  |  | 1.000* |
|  | Man | 4(50.0) | 4(50.0) |  |
|  | Female | 4(50.0) | 4(50.0) |  |
| **Nationality** |  |  |  | 0.535* |
|  | Han | 7(87.5) | 6(75.0) |  |
|  | Zhuang | 1(12.5) | 2(25.0) |  |
| **Marital status** |  |  |  | 0.809* |
| Single, divorced, widowed | | 2(25.0) | 1(12.5) |  |
|  | Married | 5(62.5) | 6(75.0) |  |
|  | Other | 1(12.5) | 1(12.5) |  |
| **BMI (kg/m^2^)** |  |  |  | 0.549 |
|  | <18.5 | 0(0.0) | 1(12.5) |  |
|  | 18.5-24 | 5(62.5) | 5(62.5) |  |
|  | 24.1-30 | 3(37.5) | 2(25.0) |  |
